# Four Jointed Box 1 Promotes Angiogenesis and Is Associated with Poor Patient Survival in Colorectal Carcinoma

**DOI:** 10.1371/journal.pone.0069660

**Published:** 2013-07-29

**Authors:** Nicole T. Al-Greene, Anna L. Means, Pengcheng Lu, Aixiang Jiang, Carl R. Schmidt, A. Bapsi Chakravarthy, Nipun B. Merchant, M. Kay Washington, Bing Zhang, Yu Shyr, Natasha G. Deane, R. Daniel Beauchamp

**Affiliations:** 1 Department of Cell and Developmental Biology, Vanderbilt University, Nashville, Tennessee, United States of America; 2 Department of Biostatistics, Vanderbilt University, Nashville, Tennessee, United States of America; 3 Department of Surgery, Vanderbilt University, Nashville, Tennessee, United States of America; 4 Department of Radiation Oncology, Vanderbilt University, Nashville, Tennessee, United States of America; 5 Vanderbilt-Ingram Cancer Center, Vanderbilt University, Nashville, Tennessee, United States of America; 6 Vanderbilt University Institute of Imaging Science, Vanderbilt University, Nashville, Tennessee, United States of America; 7 Department of Cancer Biology, Vanderbilt University, Nashville, Tennessee, United States of America; 8 Department of Biomedical Informatics, Vanderbilt University, Nashville, Tennessee, United States of America; 9 Department of Pathology, Vanderbilt University, Nashville, Tennessee, United States of America; 10 Department of Microbiology and Immunology, Vanderbilt University, Nashville, Tennessee, United States of America; Mayo Clinic College of Medicine, United States of America

## Abstract

Angiogenesis, the recruitment and re-configuration of pre-existing vasculature, is essential for tumor growth and metastasis. Increased tumor vascularization often correlates with poor patient outcomes in a broad spectrum of carcinomas. We identified *four jointed box 1* (*FJX1*) as a candidate regulator of tumor angiogenesis in colorectal cancer. *FJX1* mRNA and protein are upregulated in human colorectal tumor epithelium as compared with normal epithelium and colorectal adenomas, and high expression of *FJX1* is associated with poor patient prognosis. *FJX1* mRNA expression in colorectal cancer tissues is significantly correlated with changes in known angiogenesis genes. Augmented expression of *FJX1* in colon cancer cells promotes growth of xenografts in athymic mice and is associated with increased tumor cell proliferation and vascularization. Furthermore, FJX1 null mice develop significantly fewer colonic polyps than wild-type littermates after combined dextran sodium sulfate (DSS) and azoxymethane (AOM) treatment. *In vitro*, conditioned media from *FJX1* expressing cells promoted endothelial cell capillary tube formation in a HIF1-α dependent manner. Taken together our results support the conclusion that *FJX1* is a novel regulator of tumor progression, due in part, to its effect on tumor vascularization.

## Introduction

Angiogenesis is the process of recruiting and restructuring blood vessels from pre-existing vasculature. It is hypothesized that tumors lie dormant until undergoing an ‘angiogenic switch’ which allows enlargement of the primary tumor and a metastatic path for dissemination to other areas of the body [1]. Metastatic disease is thus reliant on vascular routes, and increased tumor angiogenesis often correlates with poor patient outcomes in a variety of carcinomas. Secreted angiogenic factors, such as vascular endothelial growth factor (VEGF), fibroblast growth factor (FGF), ephrins, netrins, and slits, are often overexpressed in cancer and provide cues that affect endothelial cell proliferation, migration, and invasion.

Increased endothelial motility and invasion has been linked to increased cyclooxygenase 2 (COX-2) expression in colon cancer cells, and up-regulation of COX-2 expression has been shown to induce autonomous production of both VEGF and FGF [2]. Elevated expression of COX-2 has been detected in colorectal carcinoma (CRC) compared to normal mucosa and correlated with increased tumor size, angiogenesis, and invasiveness, providing the rationale for development of selective COX-2 inhibitors, such as celecoxib [2]–[5]. However, subsequent clinical trials have linked the use of celecoxib to life threatening side effects such as stroke and heart attack [6], [7]. Therefore the identification of more selective therapeutic targets downstream of COX-2 signaling has become an important area of ongoing research.

In the present study, we identified *four jointed box 1*, *FJX1*, as a gene whose expression was inhibited in human rectal cancers in response to celecoxib treatment. *FJX1* gene amplification and subsequent mRNA expression has been observed in oral squamous carcinomas and in derived squamous carcinoma cell lines [8], [9]. *FJX1* mRNA expression is upregulated in ovarian tumor endothelial cells as compared to normal ovarian endothelial cells [10], [11] and thus has been suggested as a candidate tumor vasculature marker in ovarian cancer [12]. Despite these observations of increased *FJX1* mRNA expression in other carcinomas, the cellular source, biological function of FJX1 and its effects on tumor progression are unknown. Here, we report evidence that *FJX1* is a regulator of angiogenesis and higher levels of *FJX1* expression are associated with poor patient prognosis in colorectal cancer.

## Materials and Methods

### Ethics statement

Human tissues used for microarray analysis were collected and annotated according to established protocols and approved by the appropriate Institutional Review Boards (IRB) at the Moffitt Cancer Center (MCC) and Vanderbilt University (VUMC) (GSE17536 and GSE17537). Written informed consent was obtained from all patients prior to inclusion in the studies. De-identified human rectal tumor tissue for immunohistochemistry was obtained with VUMC IRB approval. All murine experiments were approved by the Vanderbilt Institutional Animal Care and Use Committee and performed in accordance with the standards of the Association of Assessment and Accreditation of Laboratory Care (AAALAC).

### Analysis of human-expression profiling

Tissue preparation, quality control, RNA isolation, and hybridization were performed as previously described [13]. The raw. CEL files of platform Affy 133 plus 2 from MCC and VUMC were combined and pre-processed using the robust multi-array average (RMA) expression measure with quantile normalization method. The Bioconductor package *Affy* (http://www.bioconductor.org/packages/release/bioc/html/affy.html) was employed. Affyprobeset 219522_at was used to compare gene expression levels of *FJX1* between different stages of colon cancer with Wilcoxon rank-sum test. Kaplan-Meier estimates for disease free and overall survival from 191 stage I–III colon cancer patients were generated using R software (http://www.r-project.org). Patients were classified as *FJX1* high and low expression groups based on a median expression value cut-off using probeset 219522_at and the log rank test was applied to determine significance.

### Celecoxib sub-group statistical analysis

The celecoxib treatment protocol was previously described [14]. Raw gene expression data (.CEL files, Affymetrix 133 plus 2 array platform) from 16 patient biopsies taken pre- and post- celecoxib treatment (32 tissue samples total), were preprocessed and normalized, as above, and expressed in log_2_ format. The bioconductor limma package was employed for array data analysis (http://www.bioconductor.org/packages/release/bioc/html/limma.html). A moderated paired t-test was used to select one hundred fifty-nine probes based on a cutoff p-value≤0.01 (un-adjusted).

### *FJX1* angiogenesis correlation analysis

WebGestalt [15] was used to identify biological processes that are significantly associated with *FJX1* expression and gene set enrichment analysis (GSEA) [16] was used to specifically test the association between *FJX1* expression and expression of other angiogenesis related genes. Two human CRC datasets GSE17536 and GSE17537 were normalized using the RMA algorithm and pairwise absolute Pearson's correlation coefficient was calculated between the *FJX1* probe set 219522_at and all other probe sets. The probe sets were ranked based on their correlation with *FJX1*. The top 500 probe sets for each dataset were subjected to the Gene Ontology biological process enrichment analysis respectively using WebGestalt. The ranked lists were analyzed using GSEA to test whether known angiogenesis genes (Gene Ontology annotation GO:0001525) were enriched at the top of the lists, identify the leading edge subset, and determine the gene set's enrichment signal [16].

### RNA extraction, RT-PCR, and qRT-PCR

RNA was extracted using the Qiagen RNeasy Kit (Qiagen) per manufacturer's instructions. RT-PCR was performed using Go-Taq (Promega) polymerase. The qRT-PCR reactions analyzing *FJX1* in human tumors were performed using superscript III reverse transcriptase (Invitrogen), SYBR Green (SA Biosciences) and analyzed on an iCycler (Bio-Rad, Inc.). Primers used : *FJX1*: 5′-CGTGCTGGCACAGTAAAGAA-3′ and 5′-TTCAAAGTTCTGGGAGGACG-3′ or 5′-AGCTGGTGGACCTAGTACAATGGA-3′ and 5′-ACTGCAGGCTGAAGAGGTTGCTTA-3′ (Integrated DNA technologies (IDT)); 18S (SAbiosciences). The qRT-PCR reactions analyzing *FJX1*, *HIF1-α*, *PMM1*, *18S* and *VEGF-A* mRNA levels in cell lines were performed using the Roche transcriptor universal cDNA master and analyzed on the Lightcycler 480 II (Roche). Primers used: *FJX1*: 5′-GAGCAGGGCTGTGACATTG-3′ and 5′-CGCTGGAACAAAGGGAGA-3′ (IDT) with probe #22 (Roche); *HIF1-α*: 5′-GGTTCACTTTTTCAAGCAGTAGG-3′ and 5′-GTGGTAATCCACTTTCATCCATT-3′ (IDT) with probe #3 (Roche); *18S*: 5′-GCAATTATTCCCCATGAACG-3′ and 5-GGGACTTAATCAACGCAAGC-3′ (IDT) with probe #48 (Roche); *VEGF-A*: 5′-CCTTGCTGCTCTACCTCCAC-3′ and 5′-CCACTTCGTGATGATTCTGC-3′ (IDT) with probe #29 (Roche); *PMM1*: 5′-TTCTCCGAACTGGACAAGAAA-3′ and 5′-CTCTGTTTTCAGGGCTTCCA-3′ (IDT) with probe #7 (Roche). Relative fold change of expression was calculated by 2^−ΔCt^.

### Plasmid construction and generation of stable lines

Full length human *FJX1* cDNA (h*FJX1*) was MYC tagged at the C terminus using the primers 5′-GATCGAATTCGGGAGCATGGGCAGGAGGATG-3′ and 5′-CTAATGCAGATCCTCTTCTGAGATGAGTTTTTGTTCAGTCCCAGACCGGCGGCCGTAC-3′, cloned into the EcoRV site of pIRES-EGFP (Clontech) and subcloned into the EcoRI site of pcDNA3.1 zeo (Invitrogen). HEK293T cells were stably transfected with pcDNA3.1 or pcDNA3.1 *hFJX1MYC*. *FJX1* cDNA was FLAG tagged by PCR amplification with the primers 5′- GATCGAATTCGGGAGCATGGGCAGGAGGATG-3′ and 5′-GATCCTCGAGAGTCCCAGACCGGCGGCCGTAC-3′ and cloned into the EcoR1 and Xho1 sites of pCMV4a (Stratagene). *hFJX1MYC* and *hFJX1FLAG* were subcloned into the EcoRI site and EcoR1/Sgf1 sites respectively of LZRS-MS-GFP (gift of Dr. Al Reynolds). Viral supernatant from HEK293T Phoenix cells transfected with LZRS-MS-GFP, LZRS-MS-GFP *hFJX1MYC*, or LZRS-MS-GFP *hFJX1FLAG* was filtered (0.45 micron) and added to target cells (KM12C, MYC; SW480, MYC; HMEC-1 and HEK293T, FLAG) for 4–8 hrs. Stable polyclonal populations were obtained via flow cytometry. HA-HIF1-α (Addgene), pcDNA3.1, and pcDNA3.1 *hFJX1MYC* were transiently transfected into HEK293T cells using effectene per the manufacturer's instructions.

### Cell culture

HEK293T, HEK293T Phoenix, and SW480 (ATCC) were cultured in RPMI 1640 (Gibco) with 10% FBS (Atlanta Biologicals), 100 U/mL pen/strep (Gibco), and 100 U/mL L-glutamine (Gibco) at 37°C with 5% CO_2_. HMEC-1 cells (F. Candl, Center for Disease Control) were cultured in MCDB131 (Gibco), supplemented with 10% FBS, 10 ng/mL epidermal growth factor (Becton-Dickson), 100 U/mL L-glutamine (Gibco), and 1 µm/mL hydrocortisone (Sigma Chemical). KM12C (Gift of Dr. Isiah Fidler) [17] were cultured in MEM (Gibco) with 10% FBS (Atlanta Biologicals), 100 U/mL pen/strep (Gibco), 100 U/mL L-glutamine (Gibco), sodium pyruvate (Gibco), non-essential amino acids (Gibco) and MEM vitamins (Gibco). Hypoxic conditions were 1% O_2_, 5% CO_2_ and 94% N_2_. MG132 (Enzo life sciences) and cycloheximide (Sigma) were used at 50 uM and 100 uM.

### Immunological detection methods and reagents

#### Generation of FJX1-specific antibodies

Nucleotides 352–1314 of human *FJX1* were amplified by PCR (5′-GATCGAATTCGTGCACGGGGGCGTCTTCTGG-3′ and 5′-GATCGTCGACCTCCCGGTGACACTAAGTCCCAGAC-3′), cloned into the EcoRI and SalI sites of pET44A (Novagen) and transformed into BL21-codon plus (DE3)-RIL (Stratagene). Transformed cells were treated with isopropyl β-D-1-thiogalactopyranoside to produce recombinant HIS tagged FJX1 protein. Recombinant HIS-FJX1 was purified using Ni-NTA beads (Invitrogen) under denaturing conditions, eluted with 2M pH = 3.0 glycine buffer, and dialyzed before immunization in rabbits in collaboration with Covance, Inc.

#### Immunoblotting

Western analyses used antibodies to: FJX1 (Covance), β-actin (Sigma Chemical), MYC 9E10 (Vanderbilt Monoclonal Antibody Core), HA (Cell signaling), and HIF1-α (Novus). De-glycosylation and de-phosphorylation were performed with Peptide N-Glycosidase F and Antarctic phosphatase (New England Biolabs) according to manufacturer's protocols.

#### Immunofluorescence

Cells were fixed in 4% paraformaldehyde for 15 minutes, blocked with 3% BSA, and incubated with primary antibodies: MYC 9E10; GM130 (BD Biosciences); FJX1 (Abcam). Secondary antibodies were: anti-rabbit DyLight 488 and anti-mouse DyLight 594 (Jackson ImmunoResearch Laboratories). Nuclei were stained with 4′,6-diamidino-2-phenylindole (DAPI) (Sigma-Aldrich). Images were captured on an Axioplan 2 upright fluorescent microscope (Carl Zeiss).

#### Immunohistochemistry

Immunohistochemistry was performed by the Vanderbilt Immunohistochemistry Core Shared Resource for Ki-67 (Vector Laboratories) and CD34 (Santa Cruz Biotechnology). Staining was quantified using the Ariol SL-50 automated slide scanner (Applied Imaging) as previously described [18]. Cross sections of CD34 stained tissue were used to determine the number of vessels per mm^2^ tissue (including perivasculature for xenograft tumors). Significance was determined using Mann-Whitney and t test.

Eleven de-identified formalin fixed and paraffin embedded human colorectal tumor tissues were obtained from the Vanderbilt Ingram Cancer Center Tissue Acquisition and Pathology Core. Xenograft tissue was also formalin fixed and paraffin embedded. Slides were sectioned at 5 µm thickness and processed as described [19] except slides were heated in 10 mM sodium citrate, pH 6.0 instead of in 100 mM Tris. Partially purified anti-FJX1 antibody was applied at a dilution of 1∶2000 and incubated overnight at 4°C. Slides were washed, incubated in 1∶500 goat anti-rabbit antibody for 30 min., washed and incubated in avidin biotin complex (Vector Labs Elite ABC kit) for 30 min and washed. Color was developed in 3, 3′ diaminobenzidine (Vector Labs) and nuclei were stained with Gill's #3 hematoxylin (Sigma). Images were taken on an Axioskop 40 upright light microscope (Carl Zeiss, Inc.).

### Collection of conditioned medium and siRNA

Conditioned media from epithelial cell lines (HEK293T, SW480, KM12C) grown in either 10% or 0% FBS, were collected at 48 hours, pelleted and transferred to a fresh tube. SW480^VEC^ or SW480^FJX1^ cells were transfected with 100 nM pooled siRNA specific to FJX1 (Qiagen), HIF1-α (Dharmacon), or non-targeting scrambled siRNA (Dharmacon), and HEK293T cells were transfected with pcDNA3.1, HA-HIF1-α or pcDNA3.1 hFJX1, replated at 48 hours post transfection, and re-fed with serum free media after 72 hours. After 96 hours, media was collected and processed as above. Medium was analyzed using the VEGF ELISA (R&D Systems) per manufacturer's instructions. ELISA data was derived from three biological replicates performed in duplicate and significance was determined using Student's t-test.

### Endothelial tube formation

HMEC1 cells in complete media were plated on top of growth factor-reduced matrigel (BD Biosciences) with or without an equal volume of conditioned media from epithelial cell lines added. Experiments were performed in triplicate at least three times. Significance was determined by Mann-Whitney, ANOVA, or Student's t test as noted.

### Cell proliferation

Cell proliferation was determined from at least 3 biological replicates performed in triplicate at each time point using the Quick Cell Proliferation Assay Kit (Biovision) per the manufacturer's instructions.

### Soft agar assay

One mL of 1.6% agarose (Sea Plaque) mixed with 2X RPM1 (1∶1) was allowed to solidify in 12 well plates for one hour at room temperature. SW480 cells (40,000 per well) in complete growth media mixed with 0.8% agarose (1∶1) were overlayed on the .8% agarose and allowed to grow for two weeks. Images and quantification were performed on GelCountTM (Oxford Optronix) according to manufacturer's protocol.

### Xenograft and mouse carcinogenesis models

One million SW480^VEC^ or SW480^FJX1^ cells were injected subcutaneously onto the flanks of athymic female nu/nu mice (Harlan Sprague Dawley). Tumor growth was monitored by taking external measurements on the animal and at the time of sacrifice (volume = 4/3πr^3^).

FJX1 -/- (KO) mice on a C57BL/6 genetic background were obtained from H. McNeil [20]. Azoxymethane (AOM, Sigma) was given intraperitoneally at 12.5 ug/g. Dextran sodium sulfate (DSS, MP Biomedicals, formula weight 36,000–50,000) was prepared in drinking water at 3%. At eight weeks of age the mice were randomized into one of four treatment groups; no treatment (KO n = 8; WT; n = 6), AOM alone (KO; n = 3; WT n = 1), DSS alone (KO n = 8; WT n = 8), or AOM and DSS (KO n = 8; WT n = 8). Mice were injected with AOM (day 1), given three cycles of DSS (days 6–10, 27–31, and 48–51) and allowed a four week recovery period [21], [22]. After experimental completion, mice were euthanized and analyzed for the number of colonic polyps. Formalin fixed paraffin embedded colonic tissue sections were then subjected to histological analysis by M.K. Washington as previously described [23] by Dieleman and colleagues. Briefly, intensity, location (mucosal, submucosal, transmural), and extent of involvement of the inflammatory infiltrate was assessed, as well as severity and extent of crypt injury.

## Results

### *FJX1* mRNA and protein expression is increased in CRC and is associated with poor patient prognosis

**Figure 1:**
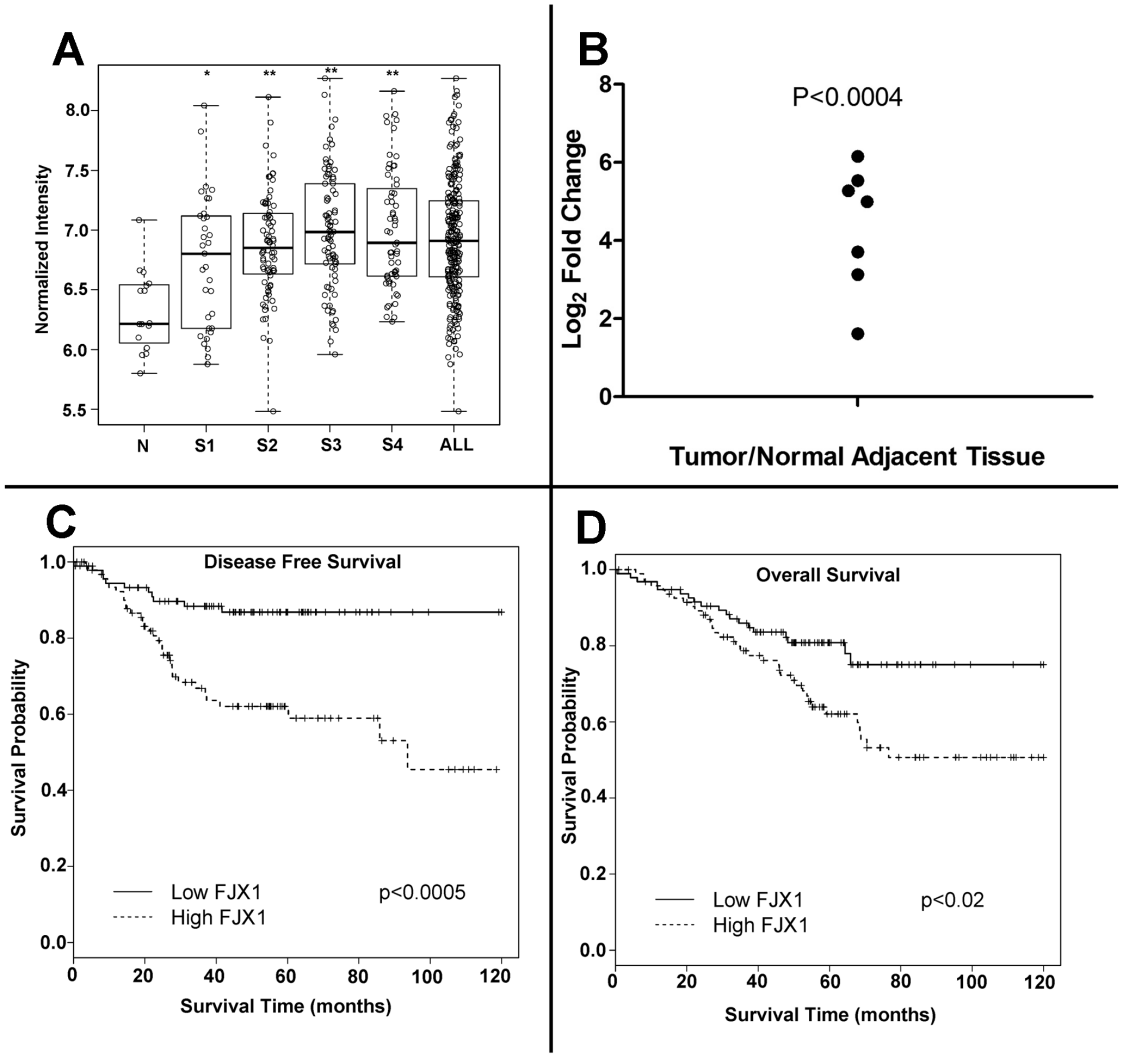
*FJX1* mRNA expression is upregulated in colorectal cancer and is associated with poor patient prognosis. (**A**) Normalized microarray-based signal intensity from *FJX1*- specific mRNA across 250 carcinoma and 16 normal (including adenoma) colorectal tissues. Significance was determined by Wilcoxon rank sum test as compared to normal. *P<0.02; **P<0.00001. N = normal; S1, S2, S3, and S4 = stage 1, 2, 3, and 4 respectively; all = stages 1–4. (**B**) Log_2_ fold change of *FJX1* specific mRNA in tumor tissue as compared to matched normal adjacent tissue as determined by qPCR. Individual datapoints represent the mean calculated values of four technical replicates for a given patient. Significance was determined using a one sample t-test. (**C/D**) Kaplan-Meier estimates relative to (**C**) disease free survival (P<0.0005, c index 0.75) and to (**D**) overall survival (P<0.02, c index 0.57) for CRC patients (stages 1–3, n = 191) separated into lower than median (solid line, n = 95) and higher than median (dashed line, n = 96) *FJX1* mRNA expression groups. Significance was determined by Log-rank test.

To identify gene expression changes in tumor tissues in response to COX-2 inhibitors, we extracted RNA from paired rectal tumor biopsies taken before and after one week treatment with celecoxib (n = 32 total; 16 pre and 16 post-treatment) [14]. Differential analysis revealed 159 expression elements mapping to 136 human genes that were significantly altered after celecoxib treatment (Raw P value<0.01, Table S1). Among those inhibited after treatment was *four jointed box one (FJX1)*, a unique gene with no known function in tumor biology. *In silico* analysis of *FJX1* mRNA expression in clinically annotated samples collected at Vanderbilt Medical Center (VUMC) and the H. Lee Moffitt Cancer Center (MCC) (see Table S2 for clinical information) revealed that *FJX1* mRNA is significantly increased across all stages of CRC as compared to normal colorectal tissue and colorectal adenomas (Figure 1A, stage 1, P<0.02; stages 2, 3 and 4, P<0.00001). There was also a significant difference between *FJX1* mRNA expression when stages 1 and 2 were compared to stages 3 and 4 (P<0.02), indicating that *FJX1* expression is further increased in more advanced stages of colon cancer.

To validate our microarray findings, we conducted quantitative RT-PCR analysis for *FJX1* mRNA and found *FJX1* mRNA expression levels were between five- and seventy-fold higher in colon cancer tissues than in normal adjacent tissue from the same patient (Figure 1B, one-sided t-test P<0.0004). Next, we examined if *FJX1* expression correlated with patient survival in a subset of stage I–III CRC patient samples from the VUMC and MCC colorectal cancer gene expression array datasets (n = 191). Samples were stratified into two groups based on lower (n = 95) and higher (n = 96) than median expression of *FJX1* and the relationship between sample *FJX1* mRNA expression and patient survival was determined by Kaplan-Meier analysis. In this retrospective analysis, patients with higher than median *FJX1* mRNA expression had significantly worse disease-free and overall survival as compared to those with lower *FJX1* expression (Figure 1, C and D). These data show that *FJX1* mRNA expression is increased in human colorectal cancer and that higher expression in tumors is associated with worse patient outcomes.

**Figure 2:**
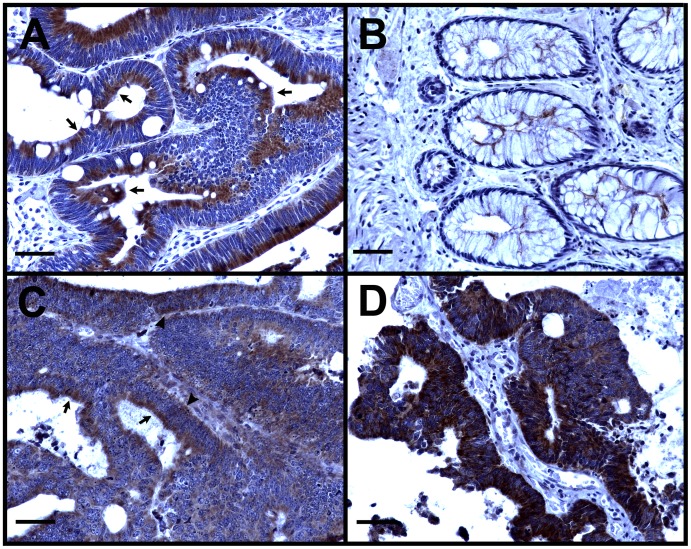
FJX1 protein expression is elevated in rectal tumors. (A) FJX1 (brown) is detected at a high level in apical cytoplasm of a well-differentiated rectal tumor. (B) Nearby adjacent normal epithelium is negative for FJX1. (C) A rectal tumor with low to moderate expression has FJX1 present mostly apically but occasionally basally. (D) In a rectal tumor showing loss of cellular polarity, FJX1 is located throughout the cytoplasm of most cells. Nuclei are counterstained in blue. Arrows indicate apical FJX1 localization; arrowheads indicate basal FJX1 localization. Scale bar = 50 µm.

By immunohistochemical analysis of colorectal tumors and adjacent normal tissues, we found that FJX1 was expressed in eight of eleven tumors at moderate to high levels that varied across the tumor in most cases, while little to no FJX1 was detected in normal mucosa (Figure 2 and data not shown). In more differentiated tumors, FJX1 was primarily located in apical cytoplasm of the epithelial cells but was also found in basal cytoplasm in less well differentiated tumors and those with greater intensity of FJX1 immunoreactivity. These data support that FJX1 protein levels are elevated in colorectal cancer tissue as compared to normal in concordance with elevated mRNA levels observed via microarray and qPCR data.

### Recombinant FJX1 localizes to the Golgi apparatus and is glycosylated and phosphorylated in both the cellular and the secreted form

**Figure 3:**
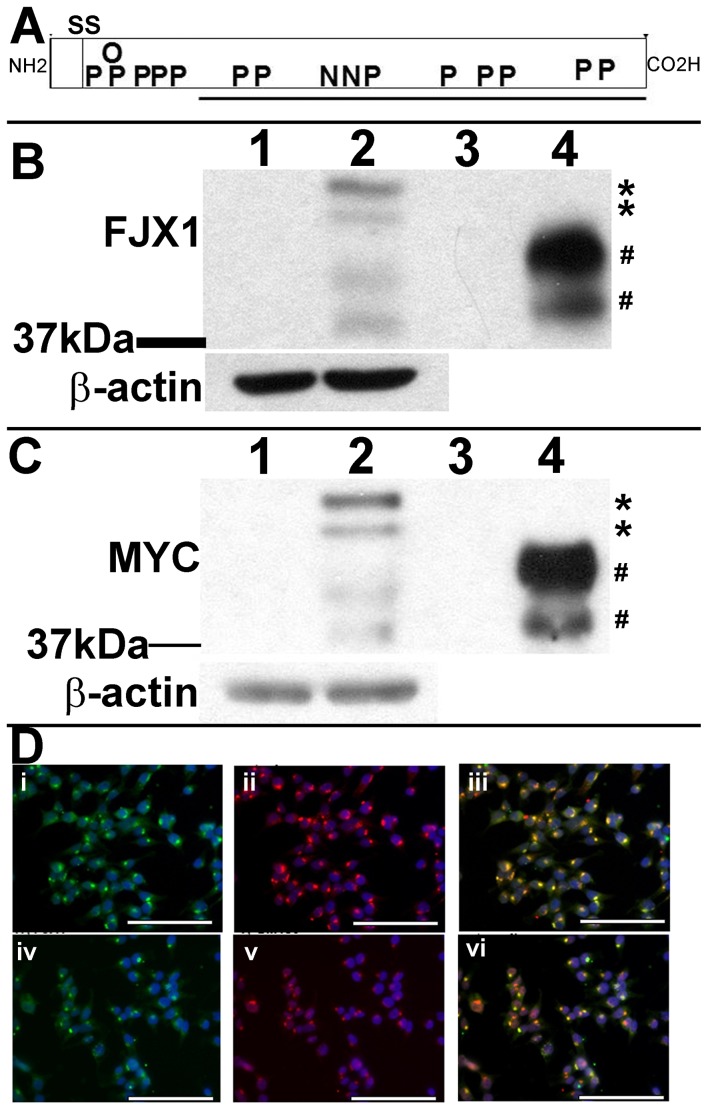
Recombinant FJX1 protein localizes to the Golgi apparatus and is secreted in HEK293T cells. (**A**) Schematic diagram of human FJX1 protein, with N-terminus (NH2) and C-terminus (CO2H) indicated. SS indicates predicted signal sequence site after amino acid 24. Approximate locations of predicted phosphorylation (P), N-linked (N) and O-linked (O) glycosylation sites are shown. Antigenic region (solid line, amino acids 118–437) used to generate antibodies is underlined. (**B, C**) Matched immunoblots of HEK293T whole cell lysate (lanes 1, 2) or conditioned media (lanes 3, 4) stably expressing vector (lanes 1,3) or MYC-tagged FJX1 (lanes 2,4) probed with (**B**) Anti FJX1 or (**C**) Anti MYC. Asterisks (*) indicate FJX1 species detected only in whole cell lysate, pound symbols (#) indicate FJX1 specific secreted forms. Anti β-actin served as the loading control for whole cell lysate. (**D**) Representative fluorescent images of HEK293T cells stably expressing MYC-tagged FJX1 dual stained for FJX1 (i, green) and MYC (ii, red) or FJX1 (iv, green) and the Golgi marker, GM130 (v, red). Nuclei were stained with 4′,6-diamidino-2-phenylindole (blue). Respective merged images are shown (iii, vi). Scale bar = 100 µm.

Since there is a paucity of published information on FJX1 function in mammalian systems, we determined the cellular and biological effects of *FJX1* expression in human cell lines. Surprisingly, screening of various cell types in culture including normal (HEK293T, YAMC, HMEC-1, HUVEC, MCF10A, RAW264.7, MCT, HT-22) and cancerous lines (SW480, SW620, HT29, HCT116, HCA7, KM12C, HCT8, DKO1, DLD1, MCF7, MDMB468, CAD, HL60, BXPC3, PANC1) failed to detect endogenous expression of FJX1. We therefore engineered a C-terminal MYC-tagged version of human *FJX1* and expressed it in human embryonic kidney cells (HEK293T) and colon cancer cells (SW480, KM12C). Based on amino acid composition, the full length human FJX1 protein (protein ID, NP_055159) has a predicted size of 48.5 kDa. There is a potential signal sequence cleavage site after amino acid 24, which would predict a processed form of approximately 46 kDa (Figure 3A). Expression of recombinant *FJX1* mRNA was confirmed by RT-PCR (Figure S1A). In whole cell lysates from MYC-tagged FJX1 transfected HEK293T^FJX1MYC^ cells, we consistently detect four FJX1 specific protein bands of approximately 48 kDa, 46 kDa, 40 kDa, and 37 kDa sizes using both an antibody we generated against recombinant FJX1 protein, (Figure 3B, lane 2) and the commercially available anti-MYC antibody (Figure 3C, lane 2). Conditioned media from HEK293T^FJX1MYC^ cells also contained 40 kDa and 37 kDa FJX1-specific bands (Figure 3, B and C, lane 4) which is consistent with observations that both *D. melanogaster* four-jointed (FJ) and *M. musculus* FJX1 proteins are secreted [24], [25]. Similar to what we observed in the HEK293T^FJX1MYC^ cells, SW480^FJX1MYC^ whole cell lysates exhibit FJX1-specific bands of approximately 46 kDa, 40 kDa, and 37 kDa, with additional bands migrating at 80 kDa and 75 kDa; again the smallest two fragments being secreted (Figure S1, B and C). All of these forms detected in SW480^FJX1MYC^ whole cell lysate and conditioned media were ablated upon treatment with siRNA specific for *FJX1* (Figure S1, B and C). Estimations of cellular proliferation in the presence or absence of serum showed no significant difference between vector and MYC-tagged *FJX1*-transduced cell lines *in vitro* (Figure S2, A and B).

Studies by Strutt and colleagues have shown that *D. melanogaster* FJ protein localizes to the Golgi apparatus [26]. Using immunofluorescence to track the intracellular pool of FJX1 in HEK293T^FJX1MYC^ we found it co-localized with the Golgi marker GM130 (Figure 3D, panels iv–vi). The specificity of FJX1 staining was confirmed by showing co-localization with MYC staining in HEK293T^FJX1MYC^ cells (Figure 3D, panels i–iii). Based upon amino acid sequence, various post-translational modifications are predicted for FJX1 protein, including two putative N-glycosylation (amino acids 248 and 277), one O-glycosylation (amino acid 53), and thirteen potential phosphorylation sites (Figure 3A). It was previously reported that the majority of exogenously expressed mouse Fjx1in HEK293T cells is a secreted protein that is sensitive to digestion with endoglycosidase H [25]. We extended this analysis by treating whole cell lysate and conditioned media from HEK293T^FJX1MYC^ cells with peptide N-glycosidase F (PNGaseF), which cleaves all polysaccharide moieties; and antarctic phosphatase, which removes phosphorylation groups. Treatment with PNGaseF resulted in a relatively uniform increase in gel electrophoresis mobility, approximately 5 kDa in size, with the upper double band collapsing into a single species (46–48 kDa to 41 kDa; 40 kDa to 35 kDa; 37 kDa to 32 kDa) suggesting that all forms of recombinant, MYC-tagged FJX1 are N-glycosylated (Figure S3, A and B, lane 1 vs. lane 3). Phosphatase treatment alone failed to significantly alter mobility of FJX1, possibly due to masking of any subtle shift by the larger effect of protein glycosylation (Figure S3, A and B, lane 1 vs. lane 2). Treatment of lysates and conditioned media with both PNGase F and phosphatase resulted in the 40 kDa and 37 kDa bands collapsing into one band, suggesting that FJX1 is phosphorylated (Figure S3, A and B, lane 1 vs. lane 4). Thus, recombinant MYC-tagged FJX1 behaves similarly to recombinant forms of the protein that have been described in both *D. melanogaster* and in *M. musculus*.

### *FJX1* expression promotes tumor growth *in vivo*

**Figure 4:**
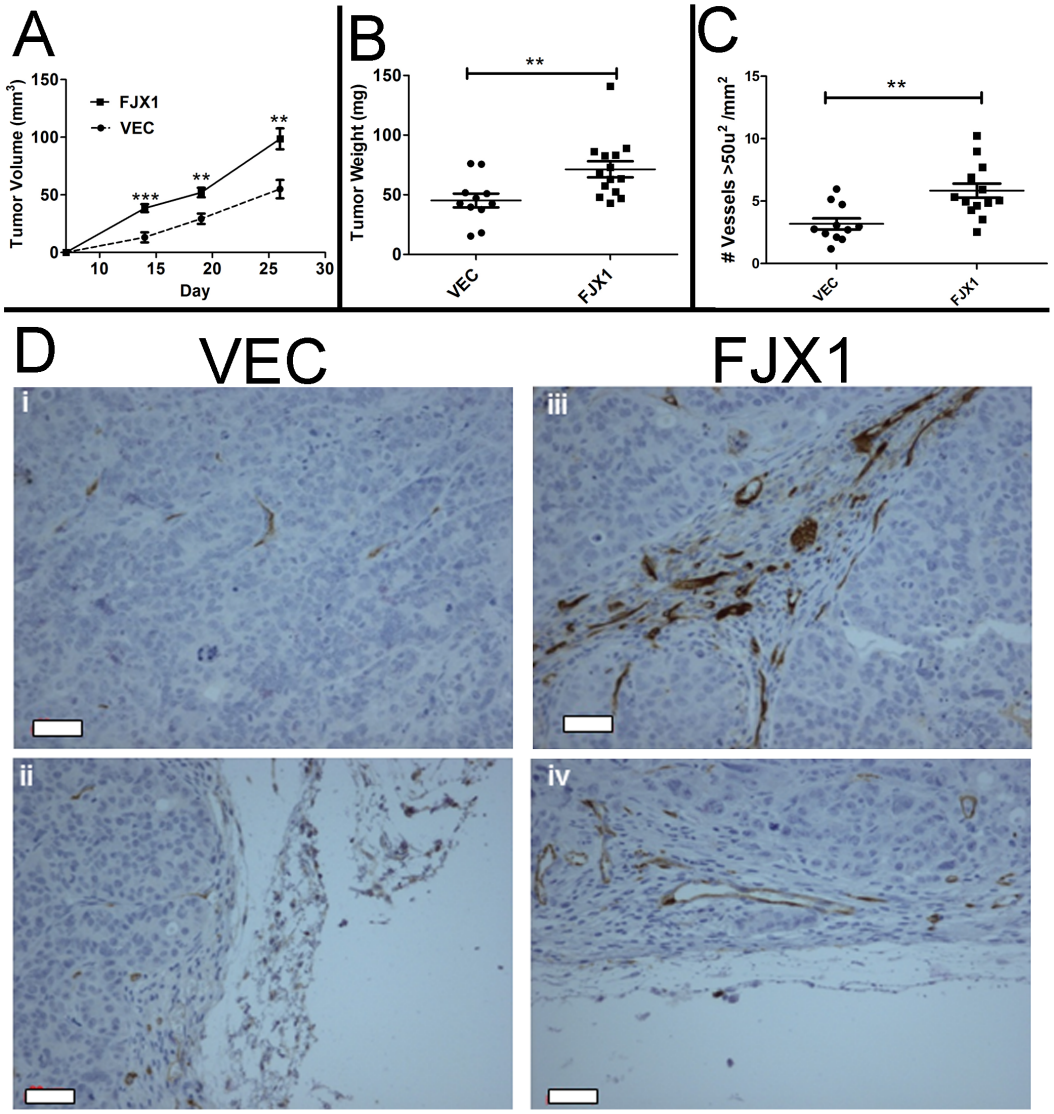
Overexpression of MYC-tagged *FJX1* in SW480 colon cancer cells promotes tumor growth *in vivo*. For all panels, VEC = SW480^VEC^ (n = 11); FJX1 = SW480^FJX1MYC^ (n = 14). (**A**) Estimation of tumor volume (mm^3^) measured *in vivo* over time. (**B**) Final tumor weight (mg) measured following removal from animal. (**C**) Number of blood vessels larger than 50 µm^2^ per mm^2^ per tumor section. Significance was determined by the Mann-Whitney test; *P<0.05, **P<0.005. Bars and whiskers represent mean and standard error of the mean respectively. (**D**) Representative light images from CD34 stained SW480^VEC^ (i–ii) or SW480^FJX1MYC^ (iii–iv) tumor sections. Scale bar = 20 microns.

To determine whether *FJX1* expression alters xenograft tumor formation *in vivo*, we next injected either SW480^VEC^ (n = 11) or SW480^FJX1MYC^ (n = 14) cells subcutaneously on the flanks of individual athymic nude mice. At 26 days post injection, both groups of mice exhibited 100% tumor incidence; however, SW480^FJX1MYC^ cells had a higher rate of tumor growth than SW480^VEC^ cells so tumors were excised for histological examination (Figure 4A). After excision, the SW480^FJX1MYC^ derived tumors were approximately twice the size (Figure S4A; average volume: 58.0 mm^3^ vs 27.1 mm^3^, P<0.0005) and with a mass 1.5 times greater than the SW480^VEC^ tumors (Figure 4B; average weight: 71.0 mg vs 45.1 mg, P<0.05). SW480^VEC^ cells produced tumors with no detectable FJX1 protein while SW480^FJX1MYC^ cells continued to produce FJX1 protein *in vivo* (Figure S4D).

We next examined whether this change in tumor growth was determined by differences in rates of proliferation or apoptosis. SW480^FJX1MYC^ tumors had significantly more Ki67-positive nuclei compared to SW480^VEC^ tumors (P<0.05), indicating an increase in the number of actively proliferating cells (Figure S4B). There was no significant difference in levels of cleaved caspase 3 (data not shown). Thus, overexpression of *FJX1* affected the rate of xenograft tumor growth due primarily to differences in the rate of tumor cell proliferation.

Since *FJX1* enhanced tumor cell proliferation *in vivo* but it had no direct effect on cell proliferation *in vitro* (Figure S2), we postulated that factors associated with the tumor microenvironment and angiogenesis contributed to this discrepancy. To test this hypothesis, tumor vasculature was measured using the endothelial marker CD34. Total CD34 staining showed a slight increase in SW480^FJX1MYC^ tumors as compared to SW480^VEC^ (data not shown). Focusing on the presence of larger vessels, that is CD34+ vessels with an area of at least 50 µm^2^, showed SW480^FJX1MYC^ tumors contained approximately twice as many of these larger vessels than SW480^VEC^ tumors per mm^2^ of tumor section (5.84 versus 3.18 respectively, P<0.005, Figure 4C, representative images in 4D). Additionally, we found no significant difference in soft agar colony formation in comparing SW480^VEC^ and SW480^FJX1MYC^ cells (Figure S4C). These data suggest that FJX1 overexpression promotes xenograft tumor growth *in vivo* by non-cell-autonomous effects, increasing recruitment of vasculature, thereby allowing increased tumor cell proliferation.

**Figure 5:**
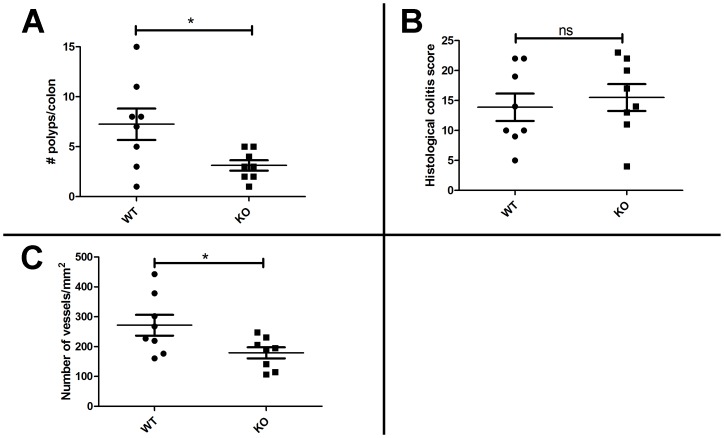
FJX1 null mice have fewer polyps than wild-type littermates in a mouse model of tumorigenesis. For all graphs WT = wild-type (n = 8); KO = FJX1 null (n = 8). (**A**) Number of colonic polyps counted in mice after treatment with AOM/DSS. (**B**) Histological colitis score as determined on hematoxylin and eosin stained colonic sections after treatment with AOM/DSS. (C) Number of blood vessels per mm^2^ of tissue section as determined on CD34 stained colonic sections after treatment with AOM/DSS. * = p<0.05. ns = not significant.

In order to determine whether endogenous expression of FJX1 has an influence on tumorigenesis, we employed a well-characterized model of inflammation/carcinogenesis using AOM and DSS to induce colonic tumors in mice [21], [22]. For this experiment we used C57BL/6 mice that were homozygous null for *FJX1* (KO, n = 8) and compared them with wild-type C57BL/6 littermates (WT, n = 8). The mice were given an initial dose of azoxymethane followed by three cycles of DSS as described in Methods. Upon gross examination, *FJX1* null mice had significantly fewer colonic polyps than the wild-type control mice (Figure 5A). We assessed the inflammation and crypt damage of formalin fixed paraffin embedded colonic sections from the *FJX1* null and WT mice as described by Dieleman et al. This method takes into account both the inflammation severity/crypt damage in combination with the percentage of tissue affected. There was no significant difference between *FJX1* null and WT mice when assessing the inflammation score, extent of inflammation, and crypt damage, which combined is known as the total histological score (Figure 5B). We then assessed the vasculature associated with *FJX1* null and WT colonic sections by quantifying CD34 stained colonic tissue sections. We found that *FJX1* null colonic sections had significantly fewer blood vessels per mm^2^ than WT mice (Figure 5C). Thus, genetic deletion of endogenous *FJX1* in mice results in inhibition of colonic tumorigenesis in association with reduced tissue angiogenesis, consistent with the increase in tumor vasculature observed in SW480^FJX1MYC^ cells grown as xenografts.

### Conditioned media from FJX1-overexpressing tumor cells increases endothelial cell tube formation *in vitro*

**Figure 6:**
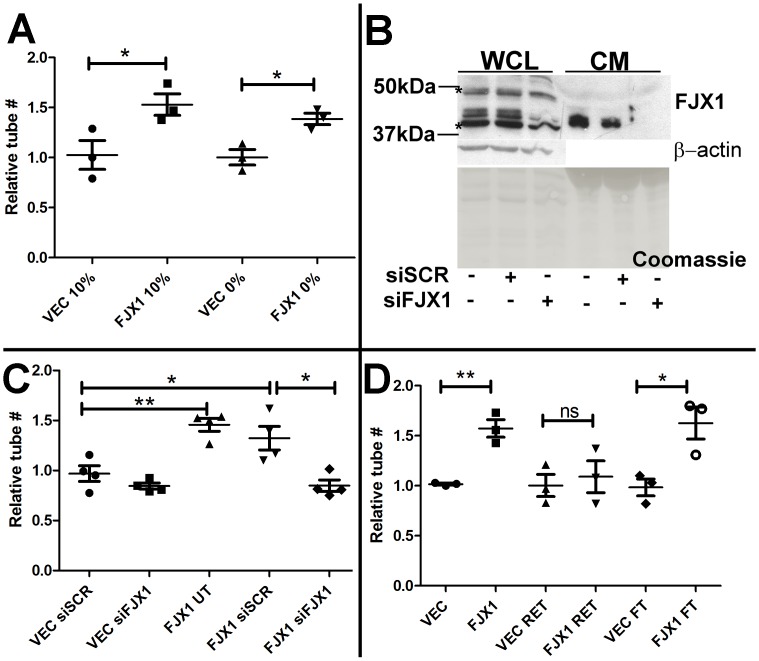
Conditioned media from SW480^FJX1^ cells enhances endothelial capillary tube formation *in vitro*. (**A**) Relative number of HMEC-1 tube structures following treatment with FJX1 conditioned media as compared with VEC conditioned media in the presence (10%) and absence (0%) of serum. (**B**) Representative immunoblot of SW480^FJX1MYC^ cells treated with siRNA specific to *FJX1* (siFJX1) or scrambled control oligonucleotide (siSCR) probed with anti-FJX1. β-actin and Coomassie stain served as loading controls for whole cell lysate (WCL) and conditioned media (CM), respectively. Asterisks (*) indicate non-specific band. (**C**) Relative number of HMEC-1 tube structures formed in the presence of conditioned media from SW480 cells treated as noted as compared to VEC untreated with siRNA (UT). (**D**) Relative number of HMEC-1 tube structures as compared with VEC after fractionation of the conditioned media. RET = retained on column; FT = flow through. For all graphs, each data point represents the value of a biological replicate and bars and whiskers represent the mean and standard error of the replicates, respectively. Significance was determined by Student's t-test; *P<0.05; **P<0.005; ns = not significant. (VEC = SW480^VEC^ and FJX1 = SW480^FJX1MYC^ conditioned media. UT = untreated. siSCR = treated with scrambled siRNA. siFJX1 = treated with FJX1 siRNA.

Since overexpression of FJX1 in the SW480 xenograft tumors resulted in increased angiogenesis, we used the widely accepted endothelial tube assay [27], [28] to test whether FJX1-conditioned media affected HMEC-1 endothelial cells *in vitro*. We found that SW480^FJX1MYC^ conditioned media significantly increased the number of endothelial tubes formed by HMEC-1 cells on Matrigel as compared to conditioned media from SW480^VEC^ cells regardless of serum concentrations (Figure 6A, P<0.05). Conditioned media from HEK293T^FJX1FLAG^ and KM12C^FJX1MYC^ cells (FJX1 immunoblot, Figure S5C) produced similar results, confirming that the endothelial phenotype is non-cell autonomous (Figure S5, A and B). Interestingly, autonomous expression of FLAG-tagged FJX1 in HMEC-1 cells also promoted tube formation *in vitro* (Figure S5D). To demonstrate that increased tube formation by SW480^FJX1MYC^ conditioned media is specifically a result of increased *FJX1* expression, we used oligonucleotides specific for *FJX1* (siFJX1) to inhibit *FJX1* expression. Inhibition of FJX1 protein expression was confirmed by immunoblotting of both whole cell lysate and conditioned media (Figure 6B). Although conditioned media from SW480^FJX1MYC^ cells untreated (UT) or transfected with scrambled siRNA (siSCR) maintained the ability to promote HMEC1 tube formation as compared to SW480^VEC^, SW480^FJX1MYC^ cells transfected with siFJX1 failed to augment tube formation (Figure 6C).

To determine if secreted FJX1 protein (approximately 40 and 37 kDa, Figure 6B) is directly responsible for the non-autonomous increase in capillary tube formation, conditioned media was fractionated using a 30,000 nominal molecular weight cut-off filter. Elimination of FJX1 protein from the flow-through fraction was confirmed by immunoblotting (Figure S5E). Interestingly, the flow-through fraction of media maintained the ability to promote HMEC-1 tube formation (Figure 6D). Thus, our data indicate that overexpression of *FJX1* by tumor cells is associated with increased secretion of other pro-angiogenic factors contained within the flow-through fraction.

### FJX1 expression causes secretion of other pro-angiogenic proteins in a HIF1-α dependent manner

In order to identify the mechanism by which FJX1 elicits pro-angiogenic activity in tumor cells, we analyzed the top 500 genes that are most highly correlated with *FJX1* expression in two human colorectal cancer datasets from VUMC and MCC. Gene Ontology enrichment analysis by WebGestalt revealed significant enrichment of angiogenesis genes in both tumor datasets. This observation was further confirmed by gene set enrichment analysis (GSEA) that directly compares the correlations between *FJX1* expression and 186 predefined angiogenic factors (GO:0001525) to those between *FJX1* expression and all other genes (Figure S6, A and B, P<0.001). This GSEA analysis identified 43 angiogenic factors with strong and consistent association with *FJX1* expression in both datasets, representing the core genes that account for the enrichment signal (the leading edge subset). These genes, including *HIF1-α*, VEGF receptors *Flt1* and *KDR*, and *VEGFC* (Table S3), suggest an association of *FJX1* and angiogenic gene expression in human colorectal cancer tumor samples.

**Figure 7:**
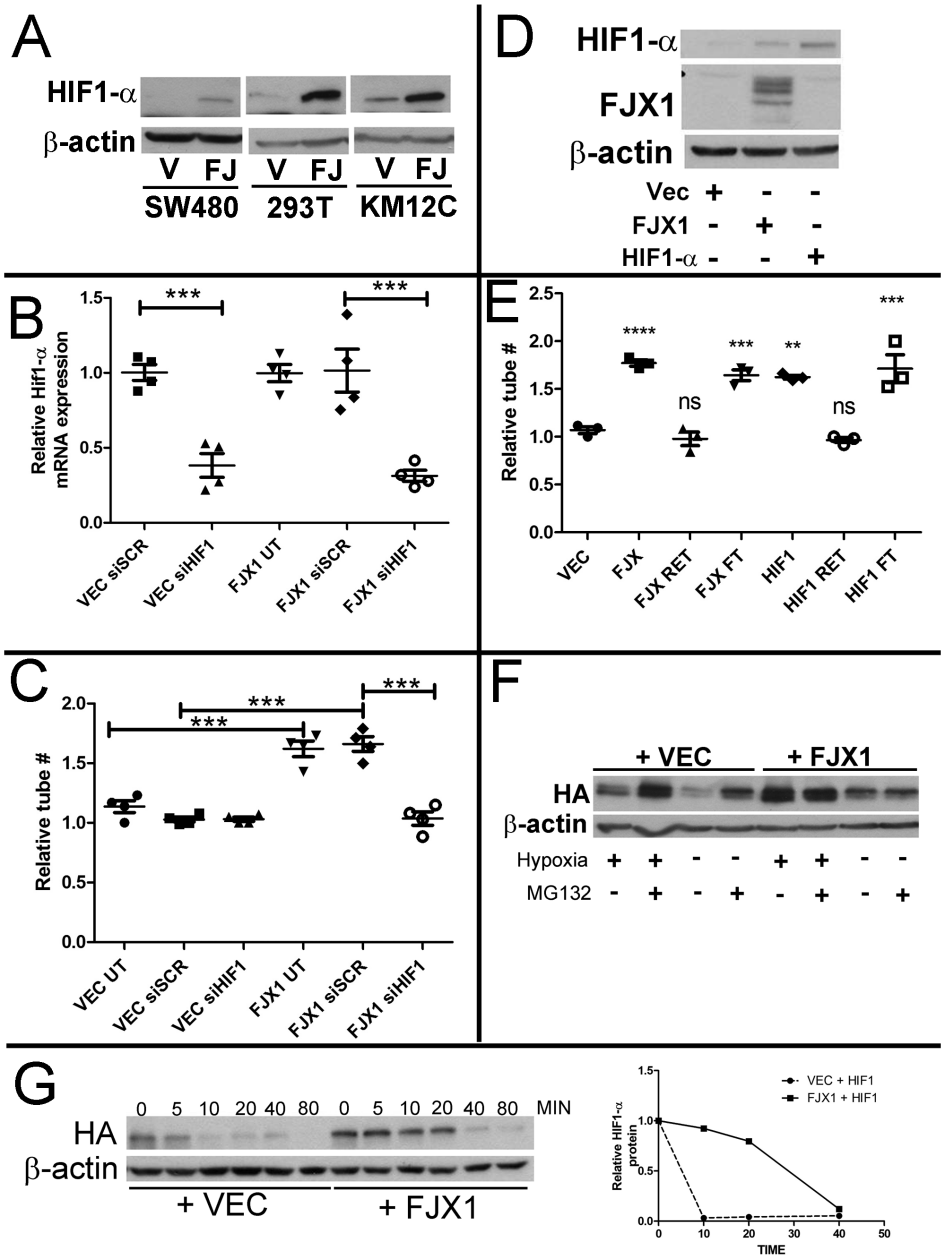
FJX1 stabilized HIF1-α contributes to increased endothelial tube formation *in vitro*. (**A**) Representative western blot of HIF1-α protein in SW480, HEK293T, and KM12C cells expressing vector (V) or FJX1 (FJ). β-actin served as the loading control. (**B**) Relative fold change in HIF1-α mRNA expression in SW480^FJX1MYC^ (FJX1) as compared to SW480^VEC^ (VEC). (**C**) Relative number of HMEC-1 tube structures as compared with SW480^VEC^ (VEC) UT after treatment with conditioned media from SW480 cell derivatives as noted. UT = untreated. siSCR = treated with scrambled siRNA. siHIF1 = treated with HIF1-α siRNA. (**D**) Representative western blot of HIF1-α and FJX1 protein in HEK293T cells transiently transfected with VEC, FJX1, or HA-HIF1-α (**E**) Relative number of HMEC-1 tube structures after treatment with conditioned media from vector, FJX1 or HIF1-α transduced HEK293T cells including fractionation of the media. RET = retained on column; FT = flow through. (**F**) Representative western blot of HEK293T cells transfected with HA-HIF1-α and VEC or FJX1 cultured in normoxia or hypoxia (4 hours) with or without treatment of MG132 (50 uM 2 hrs). (**G**) Representative western blot of HEK293T cells transfected with HA-HIF1-α and VEC or FJX1 pre-cultured in hypoxia (2 hrs) and treated with cycloheximide (100 uM) for the indicated time in minutes. Quantification is graphed on the right. (**B/C/E**) Significance was determined by ANOVA, each data point represents a biological replicate, and bars and whiskers represent mean and standard error of replicate means, respectively **P<0.005, ***P<0.0005.

Of the significant genes from the GSEA, HIF1-α is a well-characterized transcription factor that promotes the expression of several pro-angiogenic molecules. Furthermore, at least ten of the 43 genes found in the leading edge subset have been shown to be regulated by HIF1-α (Table S3) [29]–[34] [35]–[38]. Although we observed no changes in *HIF1-α* mRNA comparing vector transfected controls and *FJX1* expressing SW480, KM12C, and HEK293T cells (Figure 7B and Figure S7, A and B), we found that HIF1-α protein levels were increased in FJX1 expressing cells (Figure 7A). To test if enhanced HIF1-α expression contributed to the increased capillary tube stimulating activity in SW480^FJX1MYC^ conditioned media, we suppressed *HIF1-*α expression with siRNA specific to *HIF1-α*. Inhibition of HIF1-α was confirmed at both the mRNA (Figure 7B) and protein levels (Figure S8A). While conditioned media from SW480^FJX1MYC^ cells that were untreated or were transfected with scrambled siRNA (siSCR) maintained the ability to promote increased HMEC1 tube formation as compared to conditioned medium from SW480^VEC^ cells, SW480^FJX1MYC^ cells transfected with siHIF1-α failed to do so (Figure 7C, P<0.005). Further, inhibition of HIF1-α did not affect secreted levels of FJX1 protein (Figure S8B), supporting the conclusion that secreted FJX1 is not the direct effector of endothelial cell capillary tube formation. Rather, *FJX1* expression causes secretion of other pro-angiogenic proteins in a HIF1-α -dependent manner.

We then determined whether transient HIF1-α overexpression alone in the HEK293T cells was sufficient to induce release of the angiogenic factor into the conditioned medium. Upon transient transfection of either FJX1-MYC or HA-HIF1- α, HIF1-α protein expression was increased compared to vector transduced cells (Figure 7D) whereas HIF1-α mRNA was only increased when HA-HIF1-α was transfected (Figure S7C). Conditioned media from HEK293T cells transiently transfected with either HA-HIF1- α or FJX1-MYC stimulated HMEC-1 tube formation as compared to conditioned media from vector transduced controls (Figure 7E). Furthermore, fractionation of conditioned media using a 30,000 nominal molecular weight cut-off filter revealed that the pro-angiogenic factor was found in the flow through (Figure 7E), similar to what we observed with the SW480^FJX1MYC^ cells (Figure 6D).

VEGF-A is a transcriptional target of HIF1-α [39] and has been well characterized as an angiogenic stimulus. *VEGF-A* mRNA was upregulated in SW480^FJX1^ cells as compared with SW480^VEC^ (Figure S8C) and VEGF-A protein was increased in total SW480^FJX1MYC^ conditioned media as compared with SW480^VEC^ by ELISA (Figure S8D). However, species of VEGF-A detected by commercially available antibodies was excluded from the flow through fraction that contained the pro-angiogenic factor that promoted HMEC-1 tube formation (Figure 6D and data not shown).

To determine how HIF1-α protein levels were regulated by FJX1 we transiently expressed HA-tagged HIF1-α with and without FJX1 in HEK293T cells. When co-expressed with FJX1, HIF1-α protein levels were increased under both normoxic and hypoxic conditions (Figure 7F). Addition of the proteasome inhibitor MG132 equalized HIF1-α protein levels whether or not FJX1 was present, suggesting elevated HIF1-α protein levels were a reflection of reduced degradation rather than increased translation (Figure 7F). The addition of cycloheximide, which halts new protein translation, showed that HIF1-α protein was stabilized in the presence of FJX1 (Figure 7G). Our data therefore support a model whereby FJX1 expression can increase HIF1-α protein stability and promote secretion or release of pro-angiogenic molecules.

## Discussion

In this study, we identified *FJX1* as a candidate target of COX-2 activity associated with poor outcomes in CRC. Increased tumor levels of COX-2 have been reported in the majority of patients with CRC [3]. There is substantial experimental evidence in both mouse models and in humans documenting the effectiveness of non-steroidal anti-inflammatory drugs, particularly selective COX-2 inhibitors, in reducing both colorectal tumor formation and progression [7], [40]–[42]. Interestingly, despite our findings that rectal tumors expressed moderate to high levels of FJX1 protein and that celecoxib treatment was associated with decreased expression of *FJX1* mRNA in primary rectal cancer samples, we were unable to detect endogenous FJX1 protein expression in cultured colon cancer, human embryonic kidney or endothelial cells, and thus were unable to demonstrate a direct effect of celecoxib on *FJX1* expression. Our ability to reliably detect endogenous FJX1 in the epithelial cells of colon tumor specimens, but not in immortalized colon cancer cell lines suggests that expression of *FJX1* may require paracrine signaling or matrix interactions not supported through standard cell culture conditions. Since we also failed to detect expression of FJX1 in SW480 vector-transduced cells grown as tumor xenografts on the flanks of nude mice, but detected FJX1 protein in human colorectal tumors, it is highly likely that some component of the colonic niche is crucial in maintaining FJX1 expression in colonic cells.

We postulated that our observation that patients with higher *FJX1* mRNA expression have worse survival outcomes is related to the functional effects of *FJX1* on tumor formation. This hypothesis is supported by our experimental observations. First, mice lacking endogenous FJX1 had fewer colonic polyps after AOM/DSS treatment as compared to wild-type littermates, suggesting that FJX1 expression enhances tumor formation *in vivo*. Second, when we performed xenograft experiments in athymic mice, we found that tumors from SW480 cells transduced with *FJX1* exhibited increased size and growth as compared to vector transduced controls. In both models we found an association between vascularization and *FJX1* expression; colonic sections and tumor xenografts lacking FJX1 had fewer blood vessels. It is well recognized that without angiogenesis, tumors remain limited in both size and location, thus posing limited threat to the overall health of the individual [43]. It is likely that the correlation between high expression of *FJX1* and poor patient survival can be attributed to the pro-angiogenic effects of increased *FJX1* expression and its downstream targets.

We demonstrated that conditioned media from cells with augmented *FJX1* expression promoted endothelial capillary tube formation. This non-autonomous phenotype was maintained even upon exclusion of secreted FJX1 protein, suggesting that enhanced expression of *FJX1* is associated with increased secretion of other angiogenic factors. Since three of the cell lines in our studies, SW480, KM12C and HEK293T, do not express detectable levels of COX-2, results from these experiments argue that the effect is FJX1 specific and not due to previously described angiogenic effects of COX-2 [2]. To correlate our observations linking increased angiogenesis with enhanced *FJX1* expression (either *in vivo* or *in vitro*) we queried two human colorectal cancer datasets for association between expression of *FJX1* and known angiogenesis factors. Indeed, we found strong correlations between *FJX1* expression and expression of pro-angiogenic genes such as *HIF1-α*, *VEGF-C*, and *angiopoietin 1* and *2*. Taken together, our data suggest that *FJX1* expression may facilitate the production, release or modification of angiogenic peptides.

We detected increased HIF1-α protein in FJX1 transduced cells and experimentally linked HIF1-α levels to increased capillary tube formation. HIF1-α has been shown to induce pro-angiogenic programs through modulation of a variety of molecules including but not limited to VEGF, FLT1, ANGPT2, THBS1 and CYR61 [29]–[31], [33], [37], [38]. The ability of several known targets to either promote or hinder endothelial cell function is complex and context dependent. For example, ANGPT2 promotes endothelial sprouting in the presence of VEGF, but promotes endothelial regression in the absence of VEGF [44], [45]. Also, proteins may undergo proteolytic processing into smaller peptides that are functionally distinct from the full length form, i.e. VEGF and COL18A1 [46], [47] Although we detected increased expression of the HIF1-α-regulated VEGF-A in *FJX1* expressing cells, VEGF-A was excluded from the flow through fraction of conditioned media that contained the angiogenic stimulus associated with *FJX1* expression. This molecule may represent a smaller VEGF related fragment or peptide not detectable by available reagents, or indeed a novel HIF1-α regulated modulator of angiogenesis and is the subject of ongoing experiments.

Expression of FJX1 caused increased levels of HIF1-α through an increase in HIF1-α protein stability.

Although we initially identified a concordant relationship between *FJX1* mRNA and *HIF1-α* mRNA expression in human colonic tumors, we were only able to attribute a post-translational role of FJX1 on HIF1-α regulation *in vitro*. This discrepancy may be due to the complex interactions within the tumor microenvironment that are not supported by our *in vitro* model. Alternatively, it is possible that a common co-regulatory factor influences both *FJX1* and *HIF1-α* mRNA expression *in vivo*. Under normoxic conditions, HIF1-α protein is hydroxylated at proline residues by prolyl hydroxylases (PHD) allowing for ubiquitin mediated proteasomal degradation involving von Hippel-Lindau [48], [49]. Since PHD enzymes have an absolute requirement for molecular oxygen, hypoxic conditions inhibit PHD function and allow for HIF1-α protein stabilization. Altered mitochondrial function has also been linked to PHD activity with prevailing hypotheses being 1, that reactive oxygen species (ROS) from complex III inhibits the PHD enzymes [50], [51], or 2, that oxygen being shunted through the complex limits the availability of oxygen which is required by the PHD enzymes independently of ROS production [52], [53]. It will be interesting to determine whether FJX1 is interfering with PHD activity, perhaps by altering mitochondrial function or affecting more downstream targets that are part of the degradation complex.

Converging evidence supports the role of axon-guidance cues in both normal vasculature development (for review, [54]) and tumor associated angiogenesis [55], [56]. *FJX1* is highly expressed throughout the central nervous system during development and in the adult mouse [57], [58]. In *Fjx1* KO mice, specific subsets of hippocampal neurons exhibit either increased dendrite length or decreased arborization [58]. The observation that neuronal cues (neuropilins, ephrins, netrins, slits) are also expressed in certain tumors raised questions as to how these proteins might influence tumor development. Our observations suggest that FJX1 may represent another protein that exhibits a dual function in neuron/endothelial biology.

Through our production of FJX1-specific antibodies, we were able to characterize FJX1 localization and processing. Like *Drosophila* FJ [26], human FJX1 protein is found to localize to the Golgi apparatus where it is processed by glycosylation and phosphorylation, before secretion. Although it has been postulated that *Drosophila* FJ need not be secreted to be functional [26], murine FJX1 protein has been suggested to function as a secreted protein [58]. Here, we have experimental evidence to show that secreted FJX1 does not have a direct angiogenic-like effect *in vitro*, rather, it exerts its effect through a cell-autonomous, HIF1-α dependent mechanism. Further experimentation will be required to determine if secretion of human FJX1 protein is dispensable for function. Since FJX1 protein is a secreted molecule, it will be interesting to determine if FJX1 protein levels can be detected in patient blood or urinary samples and serve as a biomarker for colorectal cancers.

In conclusion, we provide the first evidence that *FJX1* mRNA and protein expression is increased in the epithelial cell compartment of advanced colorectal cancers. Our discovery of *FJX1* as a potential COX-2 regulated gene *in vivo* is of particular interest since numerous studies show the benefits of COX-2 inhibition in the formation and progression of CRC. The ability of FJX1 protein to enhance angiogenesis potentially explains why patients with high *FJX1* expression exhibit poor survival. Ongoing experiments are focused on further defining the mechanism by which FJX1 induces stabilization of HIF1-α.

## Supporting Information

Figure S1 **Stably expressed recombinant MYC-tagged FJX1 increases FJX1 mRNA and protein.** (A) RT-PCR amplified products of *FJX1* mRNA in HEK293T (lanes 1,2) and SW480 (lanes 3,4) cell lines stably expressing vector (lanes 1,3) or MYC-tagged *FJX1* (lanes 2,4). 18S served as the loading control. Lane 5 = reaction with out transcriptase. (B/C) Representative FJX1 protein immunoblots of vector (VEC) or MYC-tagged FJX1 (FJX1) transfected SW480 cells. (B) Whole cell lysate or (C) conditioned media from cells treated with scrambled control oligonucleotide (siSCR) or *FJX1* targeted (siFJX1) RNAi. Anti-β-actin and Coomassie stain served as loading controls for B and C, respectively. Solid arrow indicates FJX1 species detected only in whole cell lysate, dashed arrows indicate FJX1 specific secreted forms.
(PDF)

Figure S2 **Stably expressed recombinant MYC-tagged FJX1 does not affect cellular proliferation** ***in vitro*** **.** (A,B) Representative experiments of metabolized WST-1 reflecting an estimation of cellular proliferation over 5 days in (A) HEK293T and (B) SW480 cells stably expressing either empty vector (VEC) or MYC-tagged FJX1 (FJX1) grown in 0% or 10% serum as indicated. The mean values of replicates are graphed with bars indicating the standard deviation.
(PDF)

Figure S3 **Stably expressed recombinant MYC-tagged FJX1 is glycosylated and phosphorylated.** FJX1-specific immunoblots using (A) whole cell protein lysate (WCL) or (B) protein fractions from conditioned media (CM) of HEK293T cells expressing FJX1 with (+) and without (−) treatment with pngaseF and/or antarctic phosphatase.
(PDF)

Figure S4 **Overexpression of FJX1 in colon cancer cells promotes tumor growth but not colony formation.** VEC = SW480^VEC^; FJX1 = SW480^FJX1MYC^. (A) Final tumor volume measured following removal from animal. (B) Percent of Ki67 positively stained nuclei. Each data point represents quantification of an entire cross section of tumor. (C) Average number of colonies formed in soft agar. Significance was determined by Mann-Whitney. ns = not significant. *P<0.05; ***P<0.0005. Bars and whiskers represent mean and standard error of the mean respectively. (D) Representative FJX1 immunohistochemistry on SW480 xenograft tumors. Scale bar = 50 µm.
(PDF)

Figure S5 **FJX1 enhances both autonomous and non-autonomous endothelial tube formation** ***in vitro*** **.** (A/B) Relative number of HMEC-1 tube structures formed in the presence of conditioned media (CM) from (A) HEK293T or (B) KM12C cells stably transfected with empty vector (VEC) or FJX1 (FJX1) (C) Representative immunoblot of FJX1 in whole cell lysate (WCL) and conditioned media (CM) from KM12C or HEK293T cells stably expressing vector (VEC) or FJX1 (FJX1). (D) Relative number of tube structures formed by HMEC-1 cells stably transfected with empty vector (VEC) or FLAG-tagged FJX1 (FJX1). Each data point is the mean of a biological replicate. Significance was determined by Student's t test; *P<0.05; **P<0.005. Bars and whiskers represent mean and standard error of the mean respectively. (E) Representative immunoblot of FJX1 in conditioned media before fractionation (UT), in the retained (RET), and flow through (FT) fractions. VEC = SW480^VEC^ and FJX1 = SW480^FJX1MYC^ conditioned media. Coomassie served as the loading control.
(PDF)

Figure S6 ***FJX1*** **mRNA expression correlates with expression of known angiogenic factors.** Gene-set enrichment analysis of 186 defined angiogenic factors (GO:0001525) ranked by correlation from left (highest rank) to right (lowest rank) with *FJX1* expression in independent publicly available colon cancer microarray datasets (**A**) MCC and (**B**) VUMC. The enrichment score is shown by the green curve. Vertical black lines indicate the position of known angiogenic genes in the ranked list, with the density of these genes (and corresponding enrichment score) decreasing with declining correlation to *FJX1*.
(PDF)

Figure S7 ***FJX1*** **expression does not alter** ***HIF1-α*** **mRNA expression.** Relative fold change in *HIF1-α* mRNA expression in (**A**) KM12C, (**B**) HEK293T stably transfected, and (**C**) HEK293T transiently transfected with vector (VEC), FJX1 (FJX1) or HIF1-α (HIF1). Each data point is the mean of a biological replicate (**A/B**) or a technical replicate (**C**). Bars and whiskers represent mean and standard error of the mean respectively. Significance was determined by a Student's t-test. ns = not significant; **** = p<0.0001.
(PDF)

Figure S8 **Validation of HIF1-α siRNA and VEGF levels in SW480 cells.** (**A**) Representative HIF1-α immunoblot. Anti-β-actin served as the loading control. (**B**) Representative immunoblot of FJX1 in conditioned media from SW480^FJX1MYC^ cells. Coomassie stain represents loading control. (**C**) Relative fold change of VEGF-A mRNA expression as determined by qRT-PCR. Each data point is a technical replicate from one biological replicate. (**D**) Relative VEGF-A protein concentration in conditioned media. Each data point represents a biological replicate. (**C/D**) Bars and whiskers represent mean and standard error of the mean respectively. Significance was determined by a Student's t-test. VEC = SW480^VEC^ FJX1 = SW480^FJX1MYC^. siSCR = treated with scrambled siRNA. siHIF1-α = treated with HIF1-α siRNA.
(PDF)

Table S1 Affymetrix Probe ID, Entrez gene ID, p-value, gene symbol and gene name for the 159 expression elements identified as differentially expressed between pre-treated rectal tumor biopsies and celecoxib treated rectal biopsies.
(XLS)

Table S2 **Patient Sample Demographics, Pathology and Clinical Follow-up.** The numbers of patient samples used in this study are broken down by demographic, pathologic and clinical follow up characteristics. The celecoxib treatment cohort consists of 16 matched pairs of samples (pre-treatment and post-treatment) and was used to identify *FJX1* as a celecoxib responsive gene element. VUMC and MCC are publicly available datasets of fresh tumor biopsies from newly diagnosed colorectal cancer cases which had received no prior treatment and were used for establishing the association between FJX1 expression and AJCC stage and clinical outcome. Pre-treatment celecoxib samples were included in the VUMC dataset. The proportion of patient samples correlated with each demographic, pathologic and clinical characteristic is given in parenthesis. N/A = Not Applicable.
(PDF)

Table S3 **Gene symbol, gene name, and literature citation for association with HIF1-α for the top 43 genes found in the leading edge subset of both VUMC and MCC datasets after GSEA analysis.**
(XLSX)

## References

[1] Folkman J, Watson K, Ingber D, Hanahan D (1989) Induction of angiogenesis during the transition from hyperplasia to neoplasia. Nature 339: 58–61. 2469964 10.1038/339058a0

[2] Tsujii M, Kawano S, Tsuji S, Sawaoka H, Hori M, et al (1998) Cyclooxygenase regulates angiogenesis induced by colon cancer cells. Cell 93: 705–716. 9630216 10.1016/s0092-8674(00)81433-6

[3] Eberhart CE, Coffey RJ, Radhika A, Giardiello FM, Ferrenbach S, et al (1994) Up-regulation of cyclooxygenase 2 gene expression in human colorectal adenomas and adenocarcinomas. Gastroenterology 107: 1183–1188. 7926468 10.1016/0016-5085(94)90246-1

[4] Fujita T, Matsui M, Takaku K, Uetake H, Ichikawa W, et al (1998) Size- and invasion-dependent increase in cyclooxygenase 2 levels in human colorectal carcinomas. Cancer Res 58: 4823–4826. 9809985

[5] Sheehan KM, Sheahan K, O'Donoghue DP, MacSweeney F, Conroy RM, et al (1999) The relationship between cyclooxygenase-2 expression and colorectal cancer. JAMA 282: 1254–1257. 10517428 10.1001/jama.282.13.1254

[6] Kober L, Swedberg K, McMurray JJ, Pfeffer MA, Velazquez EJ, et al (2006) Previously known and newly diagnosed atrial fibrillation: a major risk indicator after a myocardial infarction complicated by heart failure or left ventricular dysfunction. Eur J Heart Fail 8: 591–598. 16507350 10.1016/j.ejheart.2005.11.007

[7] Bertagnolli MM, Eagle CJ, Zauber AG, Redston M, Solomon SD, et al (2006) Celecoxib for the prevention of sporadic colorectal adenomas. N Engl J Med 355: 873–884. 16943400 10.1056/NEJMoa061355

[8] Snijders AM, Schmidt BL, Fridlyand J, Dekker N, Pinkel D, et al (2005) Rare amplicons implicate frequent deregulation of cell fate specification pathways in oral squamous cell carcinoma. Oncogene 24: 4232–4242. 15824737 10.1038/sj.onc.1208601

[9] Jarvinen AK, Autio R, Kilpinen S, Saarela M, Leivo I, et al (2008) High-resolution copy number and gene expression microarray analyses of head and neck squamous cell carcinoma cell lines of tongue and larynx. Genes Chromosomes Cancer 47: 500–509. 18314910 10.1002/gcc.20551

[10] Buckanovich RJ, Sasaroli D, O'Brien-Jenkins A, Botbyl J, Hammond R, et al (2007) Tumor vascular proteins as biomarkers in ovarian cancer. J Clin Oncol 25: 852–861. 17327606 10.1200/JCO.2006.08.8583

[11] Lu C, Bonome T, Li Y, Kamat AA, Han LY, et al (2007) Gene alterations identified by expression profiling in tumor-associated endothelial cells from invasive ovarian carcinoma. Cancer Res 67: 1757–1768. 17308118 10.1158/0008-5472.CAN-06-3700

[12] Aird WC (2009) Molecular heterogeneity of tumor endothelium. Cell Tissue Res 335: 271–281. 18726119 10.1007/s00441-008-0672-y

[13] Smith JJ, Deane NG, Wu F, Merchant NB, Zhang B, et al (2010) Experimentally derived metastasis gene expression profile predicts recurrence and death in patients with colon cancer. Gastroenterology 138: 958–968. 19914252 10.1053/j.gastro.2009.11.005 PMC3388775

[14] Johnson JC, Schmidt CR, Shrubsole MJ, Billheimer DD, Joshi PR, et al (2006) Urine PGE-M: A metabolite of prostaglandin E2 as a potential biomarker of advanced colorectal neoplasia. Clin Gastroenterol Hepatol 4: 1358–1365. 16996805 10.1016/j.cgh.2006.07.015

[15] Zhang B, Kirov S, Snoddy J (2005) WebGestalt: an integrated system for exploring gene sets in various biological contexts. Nucleic Acids Research 33: W741–W748. 15980575 10.1093/nar/gki475 PMC1160236

[16] Subramanian A, Tamayo P, Mootha VK, Mukherjee S, Ebert BL, et al (2005) Gene set enrichment analysis: a knowledge-based approach for interpreting genome-wide expression profiles. Proc Natl Acad Sci U S A 102: 15545–15550. 16199517 10.1073/pnas.0506580102 PMC1239896

[17] Morikawa K, Walker SM, Nakajima M, Pathak S, Jessup JM, et al (1988) Influence of organ environment on the growth, selection, and metastasis of human colon carcinoma cells in nude mice. Cancer Res 48: 6863–6871. 2846163

[18] Nam KT, Varro A, Coffey RJ, Goldenring JR (2007) Potentiation of oxyntic atrophy-induced gastric metaplasia in amphiregulin-deficient mice. Gastroenterology 132: 1804–1819. 17484876 10.1053/j.gastro.2007.03.040

[19] Blaine SA, Ray KC, Anunobi R, Gannon MA, Washington MK, et al (2010) Adult pancreatic acinar cells give rise to ducts but not endocrine cells in response to growth factor signaling. Development 137: 2289–2296. 20534672 10.1242/dev.048421 PMC2889602

[20] Saburi S, Hester I, Fischer E, Pontoglio M, Eremina V, et al (2008) Loss of Fat4 disrupts PCP signaling and oriented cell division and leads to cystic kidney disease. Nat Genet 40: 1010–1015. 18604206 10.1038/ng.179

[21] Okayasu I, Ohkusa T, Kajiura K, Kanno J, Sakamoto S (1996) Promotion of colorectal neoplasia in experimental murine ulcerative colitis. Gut 39: 87–92. 8881816 10.1136/gut.39.1.87 PMC1383238

[22] Neufert C, Becker C, Neurath MF (2007) An inducible mouse model of colon carcinogenesis for the analysis of sporadic and inflammation-driven tumor progression. Nat Protoc 2: 1998–2004. 17703211 10.1038/nprot.2007.279

[23] Dieleman LA, Palmen MJ, Akol H, Bloemena E, Pena AS, et al (1998) Chronic experimental colitis induced by dextran sulphate sodium (DSS) is characterized by Th1 and Th2 cytokines. Clin Exp Immunol 114: 385–391. 9844047 10.1046/j.1365-2249.1998.00728.x PMC1905133

[24] Buckles GR, Rauskolb C, Villano JL, Katz FN (2001) Four-jointed interacts with dachs, abelson and enabled and feeds back onto the Notch pathway to affect growth and segmentation in the Drosophila leg. Development 128: 3533–3542. 11566858 10.1242/dev.128.18.3533

[25] Rock R, Heinrich AC, Schumacher N, Gessler M (2005) Fjx1: a notch-inducible secreted ligand with specific binding sites in developing mouse embryos and adult brain. Dev Dyn 234: 602–612. 16145673 10.1002/dvdy.20553

[26] Strutt H, Mundy J, Hofstra K, Strutt D (2004) Cleavage and secretion is not required for Four-jointed function in Drosophila patterning. Development 131: 881–890. 14757640 10.1242/dev.00996

[27] Kubota Y, Kleinman HK, Martin GR, Lawley TJ (1988) Role of laminin and basement membrane in the morphological differentiation of human endothelial cells into capillary-like structures. J Cell Biol 107: 1589–1598. 3049626 10.1083/jcb.107.4.1589 PMC2115245

[28] Arnaoutova I, George J, Kleinman HK, Benton G (2009) The endothelial cell tube formation assay on basement membrane turns 20: state of the science and the art. Angiogenesis 12: 267–274. 19399631 10.1007/s10456-009-9146-4

[29] Simon MP, Tournaire R, Pouyssegur J (2008) The angiopoietin-2 gene of endothelial cells is up-regulated in hypoxia by a HIF binding site located in its first intron and by the central factors GATA-2 and Ets-1. J Cell Physiol 217: 809–818. 18720385 10.1002/jcp.21558

[30] Wolf N, Yang W, Dunk CE, Gashaw I, Lye SJ, et al (2010) Regulation of the matricellular proteins CYR61 (CCN1) and NOV (CCN3) by hypoxia-inducible factor-1{alpha} and transforming-growth factor-{beta}3 in the human trophoblast. Endocrinology 151: 2835–2845. 20237132 10.1210/en.2009-1195

[31] Wan J, Chai H, Yu Z, Ge W, Kang N, et al (2011) HIF-1alpha effects on angiogenic potential in human small cell lung carcinoma. J Exp Clin Cancer Res 30: 77. 21843314 10.1186/1756-9966-30-77 PMC3174873

[32] Sanchez-Elsner T, Botella LM, Velasco B, Langa C, Bernabeu C (2002) Endoglin expression is regulated by transcriptional cooperation between the hypoxia and transforming growth factor-beta pathways. J Biol Chem 277: 43799–43808. 12228247 10.1074/jbc.M207160200

[33] Okuyama H, Krishnamachary B, Zhou YF, Nagasawa H, Bosch-Marce M, et al (2006) Expression of vascular endothelial growth factor receptor 1 in bone marrow-derived mesenchymal cells is dependent on hypoxia-inducible factor 1. J Biol Chem 281: 15554–15563. 16574650 10.1074/jbc.M602003200

[34] Elvidge GP, Glenny L, Appelhoff RJ, Ratcliffe PJ, Ragoussis J, et al (2006) Concordant regulation of gene expression by hypoxia and 2-oxoglutarate-dependent dioxygenase inhibition: the role of HIF-1alpha, HIF-2alpha, and other pathways. J Biol Chem 281: 15215–15226. 16565084 10.1074/jbc.M511408200

[35] Anelli V, Gault CR, Cheng AB, Obeid LM (2008) Sphingosine kinase 1 is up-regulated during hypoxia in U87MG glioma cells. Role of hypoxia-inducible factors 1 and 2. J Biol Chem 283: 3365–3375. 18055454 10.1074/jbc.M708241200

[36] Lai VK, Afzal MR, Ashraf M, Jiang S, Haider H (2012) Non-hypoxic stabilization of HIF-Ialpha during coordinated interaction between Akt and angiopoietin-1 enhances endothelial commitment of bone marrow stem cells. J Mol Med (Berl) 90: 719–730. 22237590 10.1007/s00109-011-0852-1

[37] Osada-Oka M, Ikeda T, Akiba S, Sato T (2008) Hypoxia stimulates the autocrine regulation of migration of vascular smooth muscle cells via HIF-1alpha-dependent expression of thrombospondin-1. J Cell Biochem 104: 1918–1926. 18384112 10.1002/jcb.21759

[38] Spinella F, Garrafa E, Di Castro V, Rosano L, Nicotra MR, et al (2009) Endothelin-1 stimulates lymphatic endothelial cells and lymphatic vessels to grow and invade. Cancer Res 69: 2669–2676. 19276384 10.1158/0008-5472.CAN-08-1879

[39] Forsythe JA, Jiang BH, Iyer NV, Agani F, Leung SW, et al (1996) Activation of vascular endothelial growth factor gene transcription by hypoxia-inducible factor 1. Mol Cell Biol 16: 4604–4613. 8756616 10.1128/mcb.16.9.4604 PMC231459

[40] Arber N, Eagle CJ, Spicak J, Racz I, Dite P, et al (2006) Celecoxib for the prevention of colorectal adenomatous polyps. N Engl J Med 355: 885–895. 16943401 10.1056/NEJMoa061652

[41] Buchanan FG, Holla V, Katkuri S, Matta P, DuBois RN (2007) Targeting cyclooxygenase-2 and the epidermal growth factor receptor for the prevention and treatment of intestinal cancer. Cancer Res 67: 9380–9388. 17909047 10.1158/0008-5472.CAN-07-0710

[42] Chan AT, Giovannucci EL, Meyerhardt JA, Schernhammer ES, Wu K, et al (2008) Aspirin dose and duration of use and risk of colorectal cancer in men. Gastroenterology 134: 21–28. 18005960 10.1053/j.gastro.2007.09.035 PMC2719297

[43] Naumov GN, Akslen LA, Folkman J (2006) Role of angiogenesis in human tumor dormancy: animal models of the angiogenic switch. Cell Cycle 5: 1779–1787. 16931911 10.4161/cc.5.16.3018

[44] Maisonpierre PC, Suri C, Jones PF, Bartunkova S, Wiegand SJ, et al (1997) Angiopoietin-2, a natural antagonist for Tie2 that disrupts in vivo angiogenesis. Science 277: 55–60. 9204896 10.1126/science.277.5322.55

[45] Lobov IB, Brooks PC, Lang RA (2002) Angiopoietin-2 displays VEGF-dependent modulation of capillary structure and endothelial cell survival in vivo. Proc Natl Acad Sci U S A 99: 11205–11210. 12163646 10.1073/pnas.172161899 PMC123234

[46] Ferrara N (2010) Binding to the extracellular matrix and proteolytic processing: two key mechanisms regulating vascular endothelial growth factor action. Mol Biol Cell 21: 687–690. 20185770 10.1091/mbc.E09-07-0590 PMC2828956

[47] Seppinen L, Pihlajaniemi T (2011) The multiple functions of collagen XVIII in development and disease. Matrix Biol 30: 83–92. 21163348 10.1016/j.matbio.2010.11.001

[48] Epstein AC, Gleadle JM, McNeill LA, Hewitson KS, O'Rourke J, et al (2001) C. elegans EGL-9 and mammalian homologs define a family of dioxygenases that regulate HIF by prolyl hydroxylation. Cell 107: 43–54. 11595184 10.1016/s0092-8674(01)00507-4

[49] Maxwell PH, Wiesener MS, Chang GW, Clifford SC, Vaux EC, et al (1999) The tumour suppressor protein VHL targets hypoxia-inducible factors for oxygen-dependent proteolysis. Nature 399: 271–275. 10353251 10.1038/20459

[50] Chandel NS, McClintock DS, Feliciano CE, Wood TM, Melendez JA, et al (2000) Reactive oxygen species generated at mitochondrial complex III stabilize hypoxia-inducible factor-1alpha during hypoxia: a mechanism of O2 sensing. J Biol Chem 275: 25130–25138. 10833514 10.1074/jbc.M001914200

[51] Guzy RD, Hoyos B, Robin E, Chen H, Liu L, et al (2005) Mitochondrial complex III is required for hypoxia-induced ROS production and cellular oxygen sensing. Cell Metab 1: 401–408. 16054089 10.1016/j.cmet.2005.05.001

[52] Hagen T, Taylor CT, Lam F, Moncada S (2003) Redistribution of intracellular oxygen in hypoxia by nitric oxide: effect on HIF1alpha. Science 302: 1975–1978. 14671307 10.1126/science.1088805

[53] Chua YL, Dufour E, Dassa EP, Rustin P, Jacobs HT, et al (2010) Stabilization of hypoxia-inducible factor-1alpha protein in hypoxia occurs independently of mitochondrial reactive oxygen species production. J Biol Chem 285: 31277–31284. 20675386 10.1074/jbc.M110.158485 PMC2951202

[54] Melani M, Weinstein BM (2010) Common factors regulating patterning of the nervous and vascular systems. Annu Rev Cell Dev Biol 26: 639–665. 19575651 10.1146/annurev.cellbio.093008.093324

[55] Nasarre P, Potiron V, Drabkin H, Roche J (2010) Guidance molecules in lung cancer. Cell Adh Migr 4: 130–145. 20139699 10.4161/cam.4.1.10882 PMC2852570

[56] Legg JA, Herbert JM, Clissold P, Bicknell R (2008) Slits and Roundabouts in cancer, tumour angiogenesis and endothelial cell migration. Angiogenesis 11: 13–21. 18264786 10.1007/s10456-008-9100-x

[57] Ashery-Padan R, Alvarez-Bolado G, Klamt B, Gessler M, Gruss P (1999) Fjx1, the murine homologue of the Drosophila four-jointed gene, codes for a putative secreted protein expressed in restricted domains of the developing and adult brain. Mech Dev 80: 213–217. 10072791 10.1016/s0925-4773(98)00218-4

[58] Probst B, Rock R, Gessler M, Vortkamp A, Puschel AW (2007) The rodent Four-jointed ortholog Fjx1 regulates dendrite extension. Dev Biol 312: 461–470. 18028897 10.1016/j.ydbio.2007.09.054

