# Supplementary figures and images for: Four Jointed Box 1 Promotes Angiogenesis and Is Associated with Poor Patient Survival in Colorectal Carcinoma
Source: PLoS One. 2013 Jul 29;8(7):e69660. doi: 10.1371/journal.pone.0069660 (PMC3726759; doi:10.1371/journal.pone.0069660)

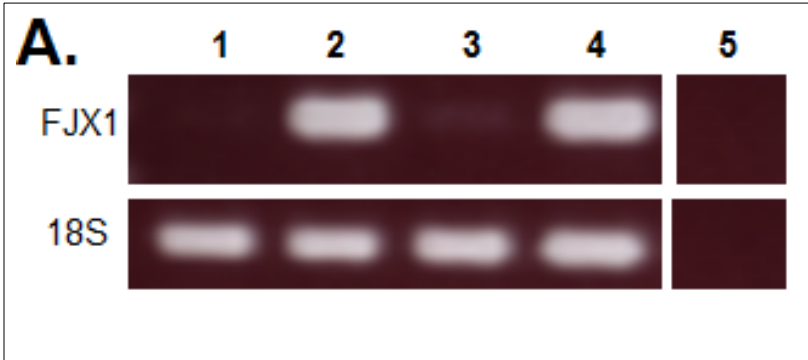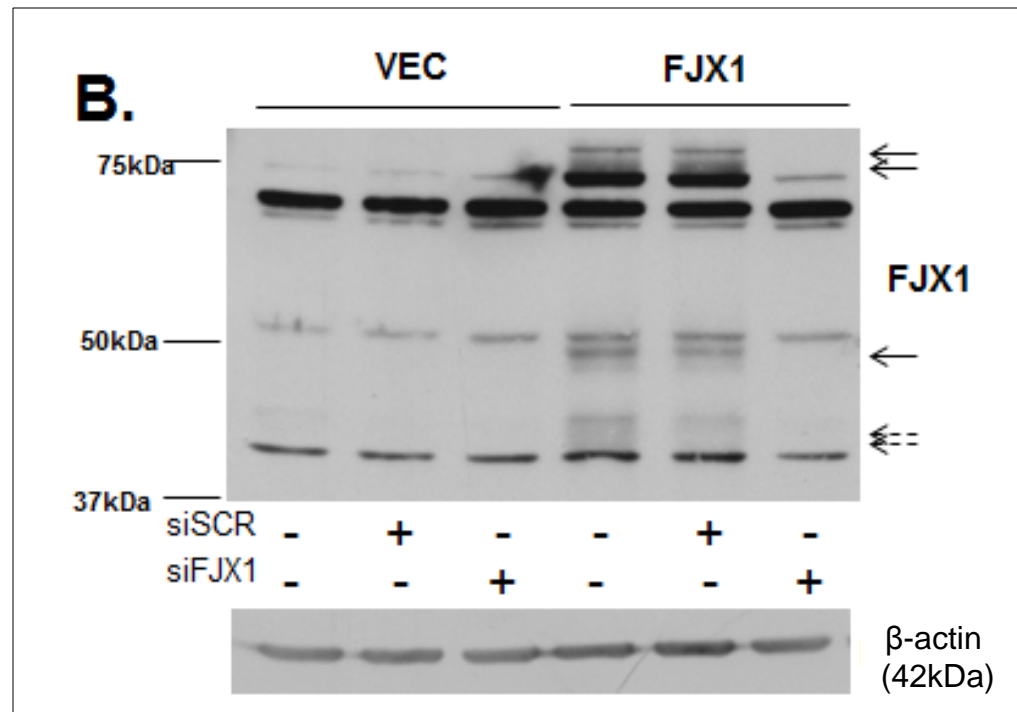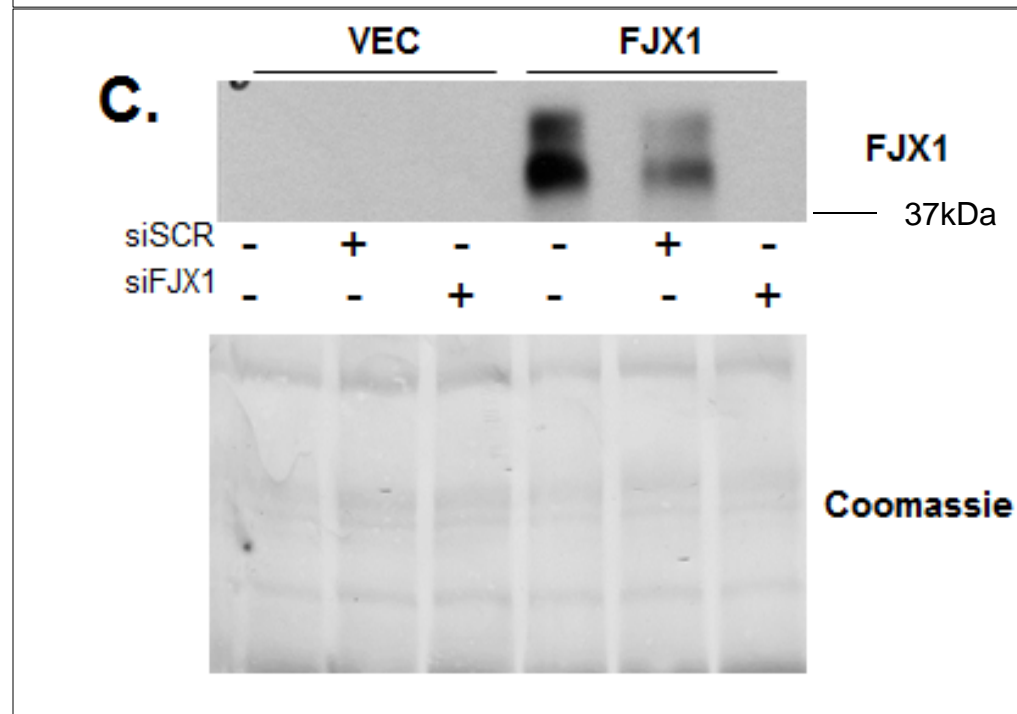

Supplement: Figure S1 — Stably expressed recombinant MYC-tagged FJX1 increases FJX1 mRNA and protein. (A) RT-PCR amplified products of FJX1 mRNA in HEK293T (lanes 1,2) and SW480 (lanes 3,4) cell lines stably expressing vector (lanes 1,3) or MYC-tagged FJX1 (lanes 2,4). 18S served as the loading control. Lane 5 = reaction with out transcriptase. (B/C) Representative FJX1 protein immunoblots of vector (VEC) or MYC-tagged FJX1 (FJX1) transfected SW480 cells. (B) Whole cell lysate or (C) conditioned media from cells treated with scrambled control oligonucleotide (siSCR) or FJX1 targeted (siFJX1) RNAi. Anti-β-actin and Coomassie stain served as loading controls for B and C, respectively. Solid arrow indicates FJX1 species detected only in whole cell lysate, dashed arrows indicate FJX1 specific secreted forms. (PDF) [file pone.0069660.s001.pdf]

**A****HEK293T**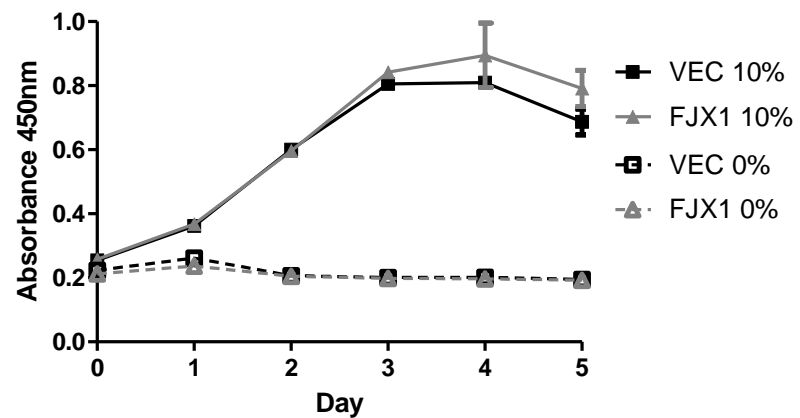**B****SW480**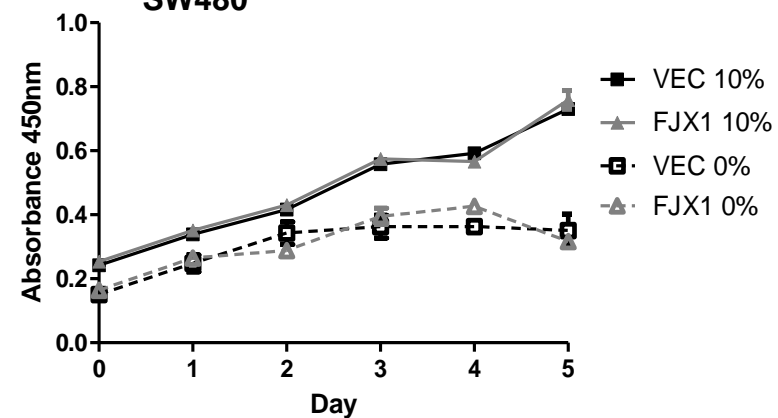

Supplement: Figure S2 — Stably expressed recombinant MYC-tagged FJX1 does not affect cellular proliferation in vitro . (A,B) Representative experiments of metabolized WST-1 reflecting an estimation of cellular proliferation over 5 days in (A) HEK293T and (B) SW480 cells stably expressing either empty vector (VEC) or MYC-tagged FJX1 (FJX1) grown in 0% or 10% serum as indicated. The mean values of replicates are graphed with bars indicating the standard deviation. (PDF) [file pone.0069660.s002.pdf]

WCL

**A**

1 2 3 4

50kDa

37kDa

Phosphatase

Pngase F

- + - +  
- - + +

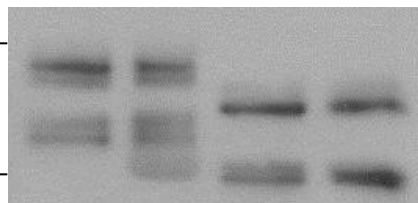

CM

**B**

1 2 3 4

50kDa

37kDa

Phosphatase

Pngase F

- + - +  
- - + +

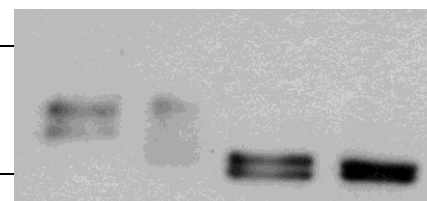

Supplement: Figure S3 — Stably expressed recombinant MYC-tagged FJX1 is glycosylated and phosphorylated. FJX1-specific immunoblots using (A) whole cell protein lysate (WCL) or (B) protein fractions from conditioned media (CM) of HEK293T cells expressing FJX1 with (+) and without (−) treatment with pngaseF and/or antarctic phosphatase. (PDF) [file pone.0069660.s003.pdf]

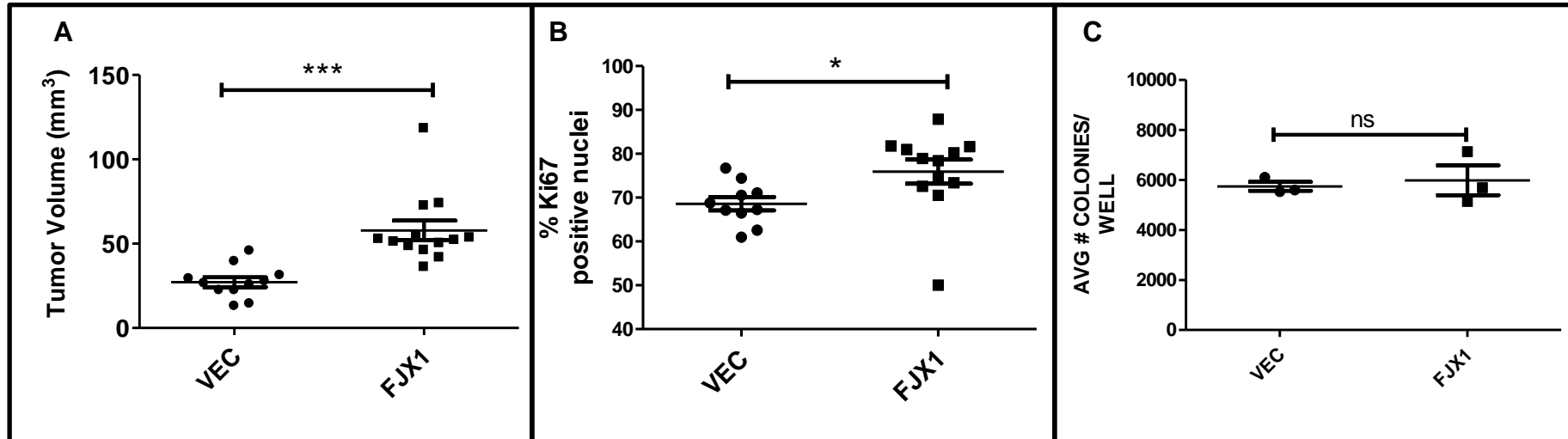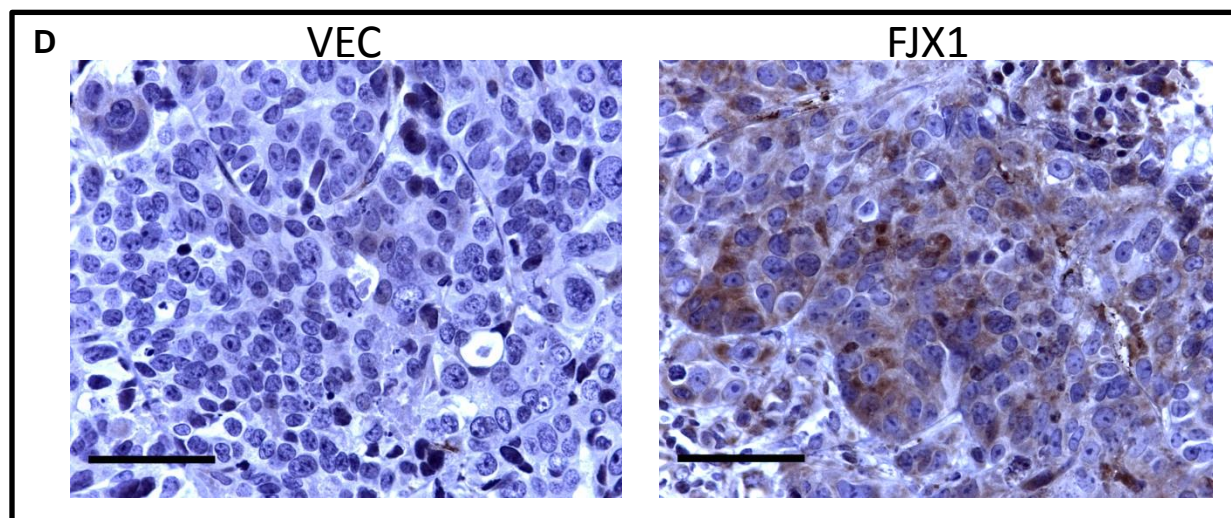

Supplement: Figure S4 — Overexpression of FJX1 in colon cancer cells promotes tumor growth but not colony formation. VEC = SW480VEC; FJX1 = SW480FJX1MYC. (A) Final tumor volume measured following removal from animal. (B) Percent of Ki67 positively stained nuclei. Each data point represents quantification of an entire cross section of tumor. (C) Average number of colonies formed in soft agar. Significance was determined by Mann-Whitney. ns = not significant. *P<0.05; ***P<0.0005. Bars and whiskers represent mean and standard error of the mean respectively. (D) Representative FJX1 immunohistochemistry on SW480 xenograft tumors. Scale bar = 50 µm. (PDF) [file pone.0069660.s004.pdf]

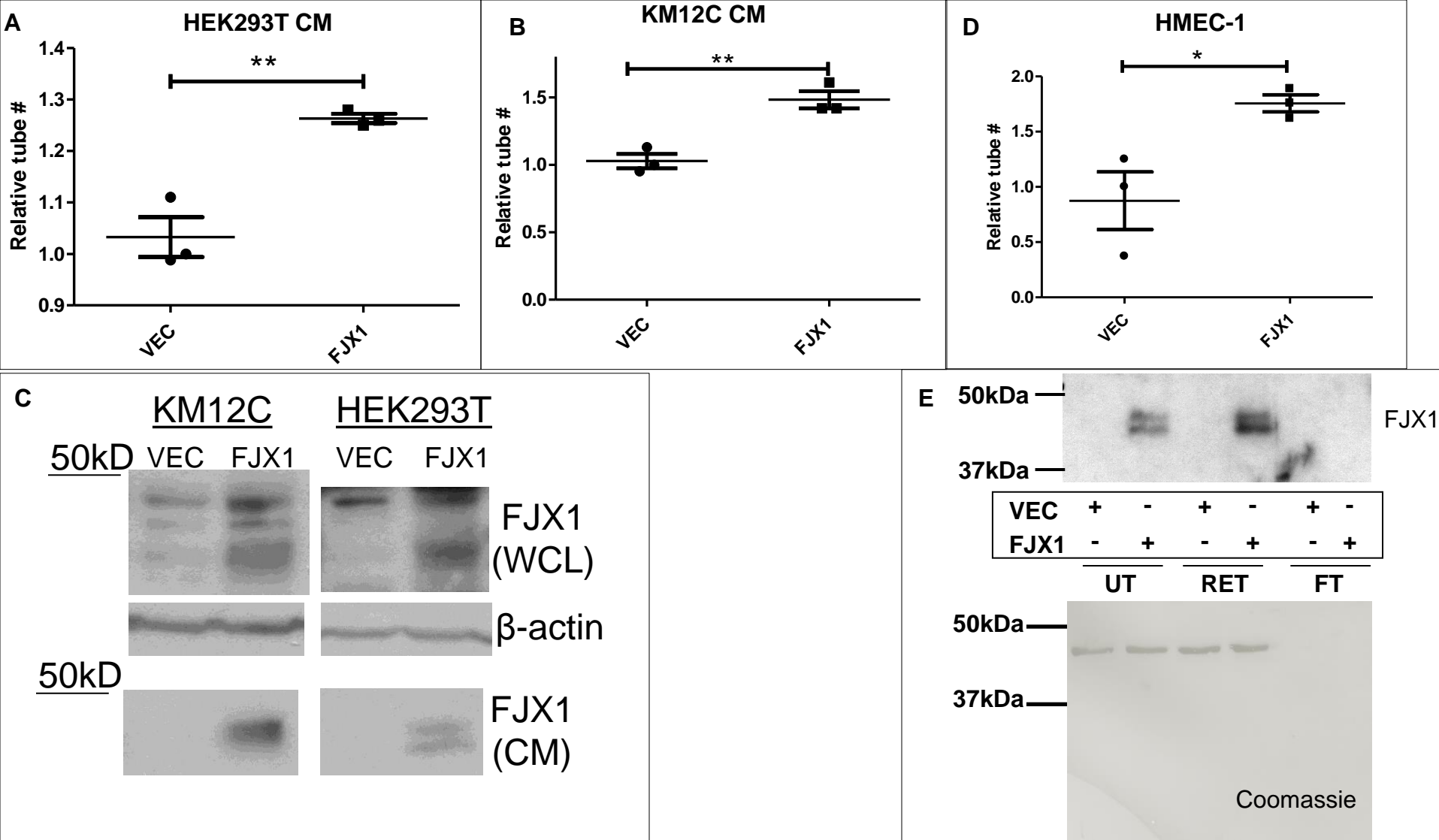

Supplement: Figure S5 — FJX1 enhances both autonomous and non-autonomous endothelial tube formation in vitro . (A/B) Relative number of HMEC-1 tube structures formed in the presence of conditioned media (CM) from (A) HEK293T or (B) KM12C cells stably transfected with empty vector (VEC) or FJX1 (FJX1) (C) Representative immunoblot of FJX1 in whole cell lysate (WCL) and conditioned media (CM) from KM12C or HEK293T cells stably expressing vector (VEC) or FJX1 (FJX1). (D) Relative number of tube structures formed by HMEC-1 cells stably transfected with empty vector (VEC) or FLAG-tagged FJX1 (FJX1). Each data point is the mean of a biological replicate. Significance was determined by Student's t test; *P<0.05; **P<0.005. Bars and whiskers represent mean and standard error of the mean respectively. (E) Representative immunoblot of FJX1 in conditioned media before fractionation (UT), in the retained (RET), and flow through (FT) fractions. VEC = SW480VEC and FJX1 = SW480FJX1MYC conditioned media. Coomassie served as the loading control. (PDF) [file pone.0069660.s005.pdf]

**A MCC**

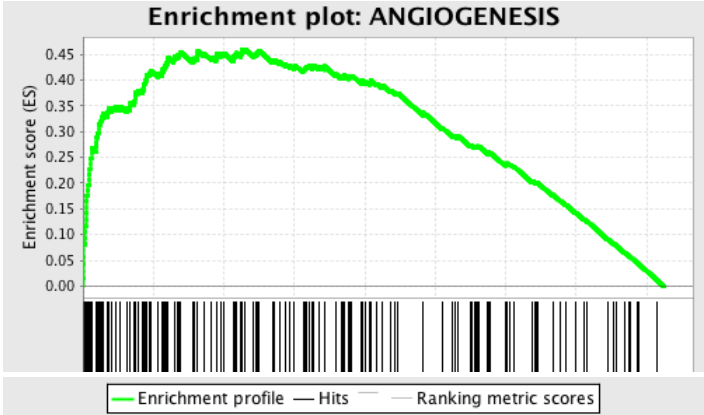

**B VUMC**

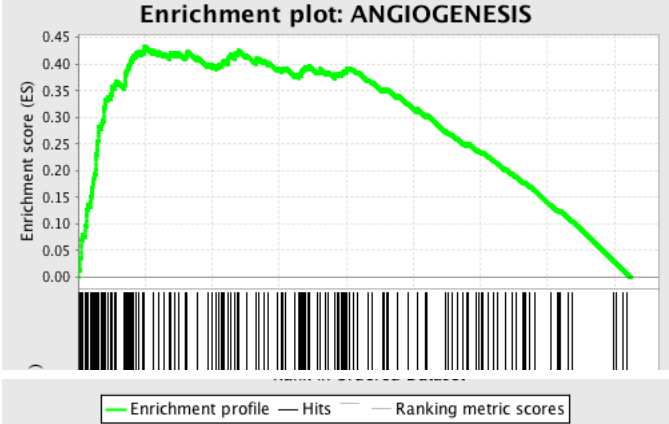

Supplement: Figure S6 — FJX1 mRNA expression correlates with expression of known angiogenic factors. Gene-set enrichment analysis of 186 defined angiogenic factors (GO:0001525) ranked by correlation from left (highest rank) to right (lowest rank) with FJX1 expression in independent publicly available colon cancer microarray datasets (A) MCC and (B) VUMC. The enrichment score is shown by the green curve. Vertical black lines indicate the position of known angiogenic genes in the ranked list, with the density of these genes (and corresponding enrichment score) decreasing with declining correlation to FJX1. (PDF) [file pone.0069660.s006.pdf]

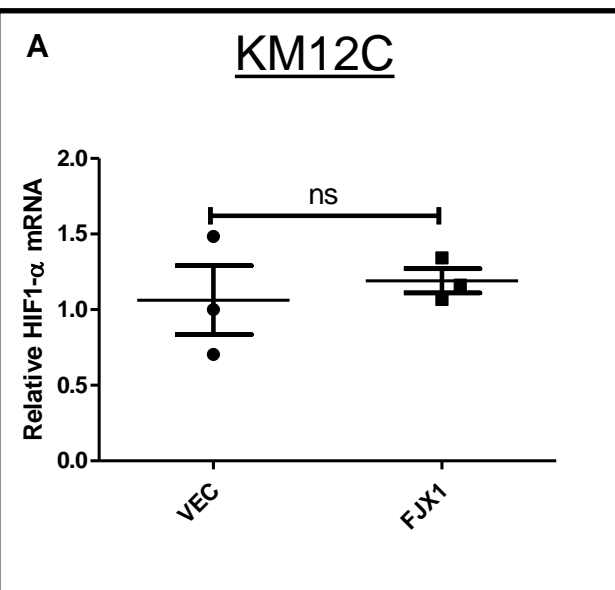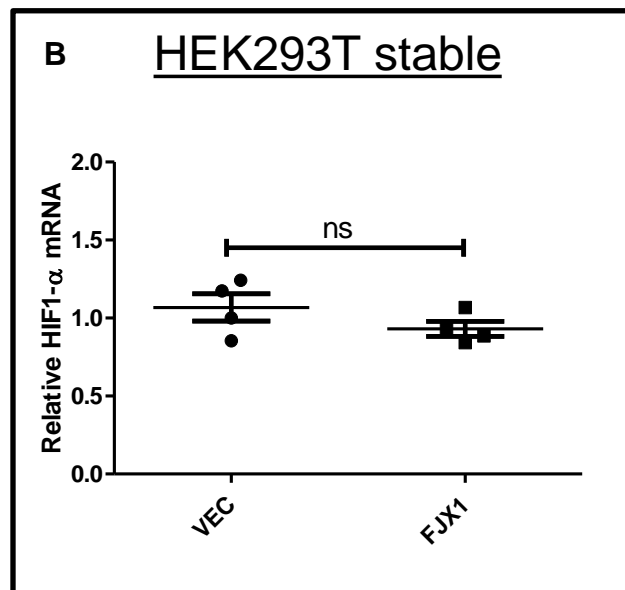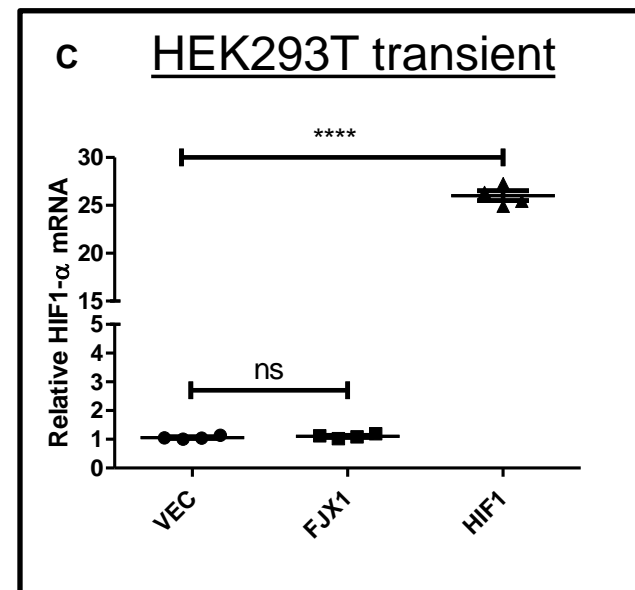

Supplement: Figure S7 — FJX1 expression does not alter HIF1-α mRNA expression. Relative fold change in HIF1-α mRNA expression in (A) KM12C, (B) HEK293T stably transfected, and (C) HEK293T transiently transfected with vector (VEC), FJX1 (FJX1) or HIF1-α (HIF1). Each data point is the mean of a biological replicate (A/B) or a technical replicate (C). Bars and whiskers represent mean and standard error of the mean respectively. Significance was determined by a Student's t-test. ns = not significant; **** = p<0.0001. (PDF) [file pone.0069660.s007.pdf]

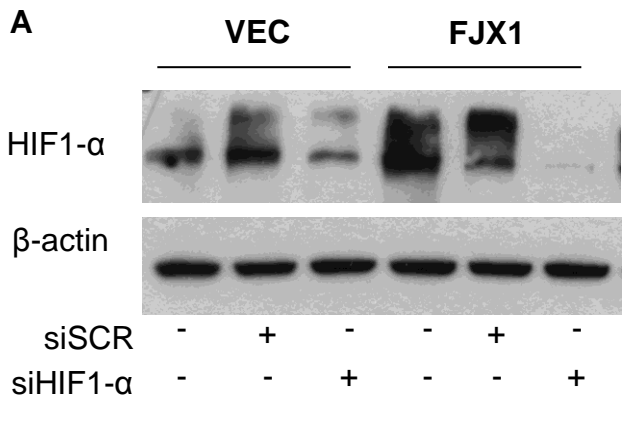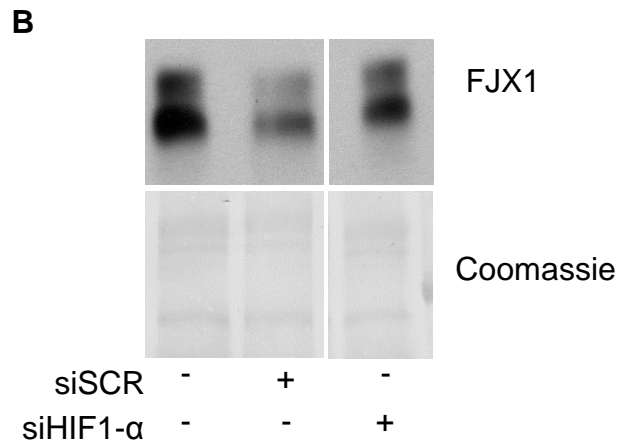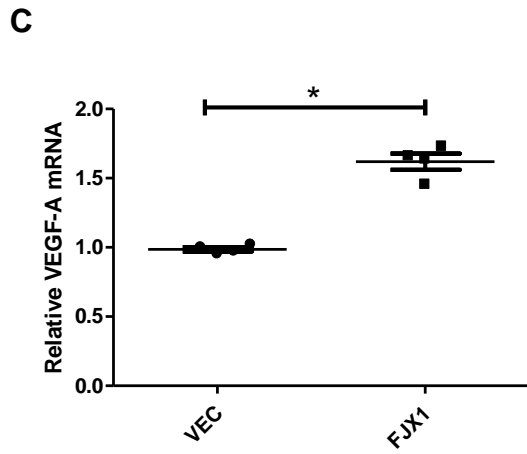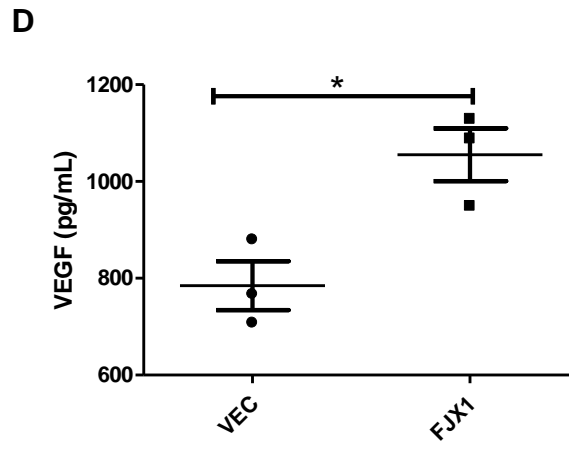

Supplement: Figure S8 — Validation of HIF1-α siRNA and VEGF levels in SW480 cells. (A) Representative HIF1-α immunoblot. Anti-β-actin served as the loading control. (B) Representative immunoblot of FJX1 in conditioned media from SW480FJX1MYC cells. Coomassie stain represents loading control. (C) Relative fold change of VEGF-A mRNA expression as determined by qRT-PCR. Each data point is a technical replicate from one biological replicate. (D) Relative VEGF-A protein concentration in conditioned media. Each data point represents a biological replicate. (C/D) Bars and whiskers represent mean and standard error of the mean respectively. Significance was determined by a Student's t-test. VEC = SW480VEC FJX1 = SW480FJX1MYC. siSCR = treated with scrambled siRNA. siHIF1-α = treated with HIF1-α siRNA. (PDF) [file pone.0069660.s008.pdf]
