# Supplementary material for: Four Jointed Box 1 Promotes Angiogenesis and Is Associated with Poor Patient Survival in Colorectal Carcinoma
Source: PLoS One. 2013 Jul 29;8(7):e69660. doi: 10.1371/journal.pone.0069660 (PMC3726759; doi:10.1371/journal.pone.0069660)
Supplement: Table S2 — Patient Sample Demographics, Pathology and Clinical Follow-up. The numbers of patient samples used in this study are broken down by demographic, pathologic and clinical follow up characteristics. The celecoxib treatment cohort consists of 16 matched pairs of samples (pre-treatment and post-treatment) and was used to identify FJX1 as a celecoxib responsive gene element. VUMC and MCC are publicly available datasets of fresh tumor biopsies from newly diagnosed colorectal cancer cases which had received no prior treatment and were used for establishing the association between FJX1 expression and AJCC stage and clinical outcome. Pre-treatment celecoxib samples were included in the VUMC dataset. The proportion of patient samples correlated with each demographic, pathologic and clinical characteristic is given in parenthesis. N/A = Not Applicable. (PDF) [file pone.0069660.s010.pdf]

	Celecoxib Treatment	VUMC + MCC
Total number of patients	N=16	N=250
Mean age +/- SD	60.0 +/- 10.00	65.0 +/- 13.26
Male	8 (50.0%)	132 (52.8%)
Stage I	4 (25.0%)	33 (13.2%)
Stage II	4 (25.0%)	76 (30.4%)
Stage III	8 (50.0%)	82 (32.8%)
Stage IV	0 (0.0%)	59 (23.6%)
Median follow-up (months)	N/A	42.7
Deaths	N/A	84 (33.6%)
Caucasian	14 (88.0%)	215 (86.0%)
African-American	2 (12.0%)	15(6.0%)
Unknown/Other	0.0	20 (8.0%)
